# Expression variability of co-regulated genes differentiates *Saccharomyces cerevisiae *strains

**DOI:** 10.1186/1471-2164-12-201

**Published:** 2011-04-20

**Authors:** Laura Carreto, Maria F Eiriz, Inês Domingues, Dorit Schuller, Gabriela R Moura, Manuel AS Santos

**Affiliations:** 1 RNA Biology Laboratory, CESAM & Department of Biology, Universidade de Aveiro, 3810-193 Aveiro, Portugal; 2 BIOCANT, Centro de Inovação em Biotecnologia, Parque Tecnológico de Cantanhede, Núcleo 04, Lote 3, 3060-197 Cantanhede, Portugal; 3 Centro de Biologia Molecular e Ambiental (CBMA) Universidade do Minho, Braga, Portugal

## Abstract

**Background:**

*Saccharomyces cerevisiae *(Baker's yeast) is found in diverse ecological niches and is characterized by high adaptive potential under challenging environments. In spite of recent advances on the study of yeast genome diversity, little is known about the underlying gene expression plasticity. In order to shed new light onto this biological question, we have compared transcriptome profiles of five environmental isolates, clinical and laboratorial strains at different time points of fermentation in synthetic must medium, during exponential and stationary growth phases.

**Results:**

Our data unveiled diversity in both intensity and timing of gene expression. Genes involved in glucose metabolism and in the stress response elicited during fermentation were among the most variable. This gene expression diversity increased at the onset of stationary phase (diauxic shift). Environmental isolates showed lower average transcript abundance of genes involved in the stress response, assimilation of nitrogen and vitamins, and sulphur metabolism, than other strains. Nitrogen metabolism genes showed significant variation in expression among the environmental isolates.

**Conclusions:**

Wild type yeast strains respond differentially to the stress imposed by nutrient depletion, ethanol accumulation and cell density increase, during fermentation of glucose in synthetic must medium. Our results support previous data showing that gene expression variability is a source of phenotypic diversity among closely related organisms.

## Background

*Saccharomyces cerevisiae *exists in diverse ecological niches across the globe and can be found in natural habitats associated with fruits, trees and insect guts [1-5], and is also being used by humans for millennia for brewing, bread and wine production. Recently, wild type strains have been isolated from infections of immunocompromised individuals [6], raising concerns about their virulence potential. Large scale genome comparisons [7,8] have demonstrated that the genetic diversity among strains is high and involves single nucleotide polymorphisms (SNPs), insertions and deletions (indels), gene copy number and gene content variation. The occurrence of independent adaptation events, together with its domestication and dispersion associated to bread, beer and wine production [9], have been proposed to explain the variability observed among wild type strains. Interestingly, clinical strains belong to unrelated phylogenetic lineages, raising the hypothesis that pathogenic traits are ubiquitous in yeast.

Genome expression plasticity is important in yeast for adaptation to new environments. Genes whose expression is associated to phenotypic variability - such as those encoding proteins involved in amino acid biosynthesis and transport, sulphur and nitrogen assimilation, and protein degradation [10-12] - are strongly regulated under environmental stress [13,14], with implications for fitness under environmentally dynamic conditions. Quantitative trait loci (QTL) (see references [15-24]) can explain inter-strain phenotypic diversity arising from subtle alterations in gene sequences, in particular if they alter gene expression [23,25-28]. QTLs may even be responsible for stochastic "noise" in gene expression which increases phenotypic variability in an unpredictable manner [29]. However, the role of gene expression variability in evolution and cell biology is still poorly understood.

DNA microarrays enable the analysis of global patterns of gene expression and allow for identification of gene expression variability. However, few studies have so far focused on the comparative analysis of gene expression patterns among wild type yeast strains. Furthermore, comparative gene expression studies have been carried out in exponentially growing cultures [10-12,30-32] and studies concerning gene expression diversity upon entry or during stationary phase [33] are still scarce. An important caveat of stationary phase is the low transcription and translational rates [34-36] which complicate comparative transcriptome analysis. However, quiescence is likely the most common metabolic state in nature [33] and it is essential for yeast survival and evolutionary processes. Therefore, comparative transcriptomics studies of quiescent cells are needed to understand adaptation potential in environmental settings.

In a previous study, we have characterized the genome variability of 16 yeast strains of laboratory, commercial, environmental and clinical origin, using comparative genome hybridization on array (aCGH) [37]. The data showed that Ty element copy number differentiated environmental and commercial strains from other types of strains, whereas sub-telomeric instability was associated with clinical and laboratorial strains. Our data corroborated others results showing that those genome rearrangements are important sources of genetic diversity among natural populations of yeast [38-40]. Our study also highlighted variability in copy number of genes involved in amino acid and sugar metabolism, among others, which are relevant for environmental adaptation. The molecular mechanisms responsible for gene copy number alterations are not yet clear, but such alterations should affect transcript abundance and may explain, at least in part, gene expression differences between strains [41].

In the present study, we have used five yeast strains isolated from different environmental biotopes, one clinical and one laboratorial reference strain (S288C), to investigate transcriptome variability in yeast. Transcriptome profiles were obtained at different growth time points of exponential, diauxic shift and stationary phase states. Since environmental strains were isolated from vineyards, fermentation in synthetic wine must was chosen for this study. Under these conditions, yeast cells have to cope with multiple stresses, including high osmotic pressure, low pH, nutrient deprivation, starvation and high ethanol concentration. Since changes in nutritional, environmental and physiological conditions trigger highly coordinated transcriptional reprogramming of master regulatory pathways [42], our study takes advantage of these changes in gene expression to evaluate for the first time variability among strains in a continuously changing environment.

We have identified variable genes and metabolic pathways affected by gene expression variability and we demonstrate that metabolic reprogramming at the transition between fermentation and respiration enhances variability in gene expression. Our data indicates that metabolic transitions expose variability and are important time points to study gene expression noise and for phenotypic differentiation of yeast strains. Environmental isolates associated to wine fermentation showed lower levels of expression of genes involved in the responses to stress and nutrient depletion, in particular during late stages of fermentation, suggesting that they are better adapted to the stress imposed by this particular environmental condition.

## Results

### Characterization of yeast strains

**Figure 1:**
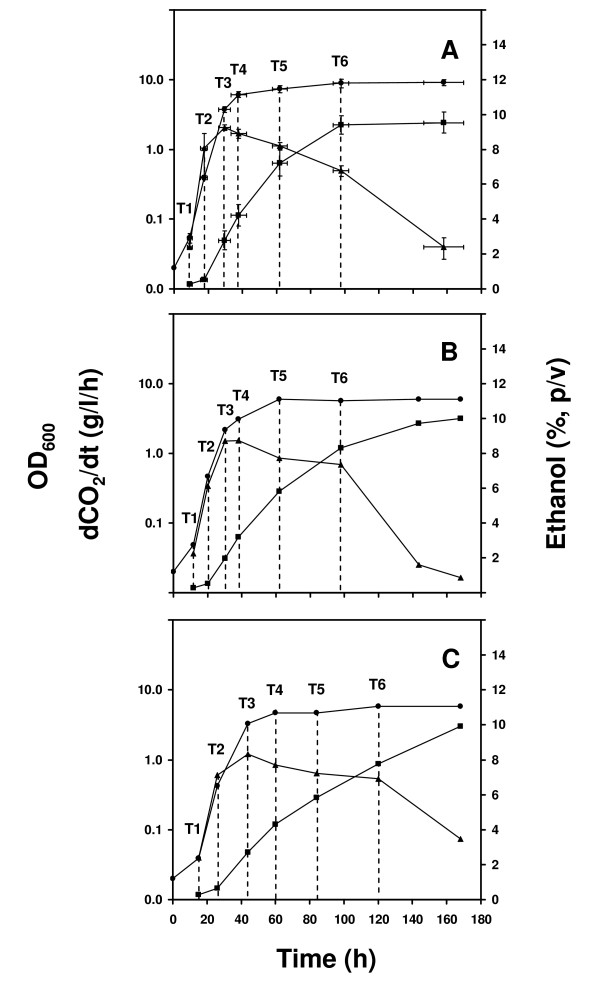
**Fermentation profiles of wild-type yeast strains**. The fermentation profiles of the seven yeast strains used in this study are compared in respect to cell growth (OD_600_; -●-), CO_2 _production rate (dCO_2_/dt; -▲-) and ethanol concentration (%, p/v) (-■-): A) Averaged data for strains 06L3FF02, 06L6FF20, AEB Fermol Rouge, Lalvin ICV D254 and Lalvin EC-1118 (bars indicate standard deviations from the mean value represented); B) strain J940047; C) strain S288C. Vertical dashed lines identify the time points used for transcriptome profiling (see text for details).

In this study, we have used two *S.cerevisiae *strains isolated from vineyards of the Bairrada wine region in Portugal, (06L3FF02 and 06L6FF20), three commercial wine strains (AEB Fermol Rouge, Lalvin EC-1118 and Lalvin ICV D254), a strain isolated from a patient suffering from opportunistic fungal infections (J940047) plus the laboratory strain S288C. Fermentation in synthetic wine must occurred with similar exponential growth rate (doubling time of 3.3 ± 0.1 hours) and comparable final biomass and ethanol concentrations (approximately 10% (p/v) for all strains (Figure 1). The environmental and commercial strains reached maximum ethanol concentration after 96 h of fermentation, while the clinical and laboratorial strains took ~170 h. The fermentation profiles of the environmental and commercial strains were identical and therefore averaged (Figure 1A). Strain J940047 had a slightly delayed fermentation onset which did not affect the profile (Figure 1B). All fermentation stages were delayed in strain S288C relative to the wild-type strains (Figure 1C) but all strains showed similar cell viability (90-100%) throughout the monitored fermentation time (170 hours). The viability decline in stationary phase was strain-dependent and strain Lalvin EC-1118 lost its capacity to form colony forming units earlier than the other strains, after 10 days of fermentation (Additional file 1).

The mRNA samples for transcriptome profiling were collected at six time points, representing the beginning of fermentation (T1, after one cell division post-inoculation, allowing for the initial lag period and the doubling of the OD_600 _of the culture), mid-exponential growth (T2; OD_600 _~0.5), growth stage transition (T3, maximum rate of CO_2 _production) and during early, intermediate and late stationary phase (T4, T5 and T6, respectively) (Figure 1). Ethanol concentration was ~7% (p/v) at T5 (p/v) and ~9% (p/v) at T6 for the environmental and commercial strains and ~1% (p/v) lower for strains J940047 and S288C at the same time points.

### Transcriptome profiles

We used oligonucleotide (70 mer) DNA microarrays targeting the ORFeome sequences of the laboratory strain S288C for comparative transcriptome analysis. With these arrays, the putative divergence of the genomic sequence of the investigated strains relatively to that of S288C [7,8] should not originate hybridization biases, since it is below the limit for unspecific target detection with probes of this length [43]. A common reference sample was obtained from strain S288C grown to mid-exponential phase in the same growth conditions and was used for co-hybridization with all samples. As expected from the reduction in transcription that occurs in yeast stationary phase [44], total RNA extracts obtained from samples T4, T5 and T6 produced lower amount of cDNA, compromising the labelling of the samples for microarrays and raising questions about the representativeness of the data obtained from eventual pooling of the cDNA synthesis reactions. To overcome this problem, mRNA enriched samples were used to synthesize cDNA for microarray analysis and *in vitro *synthesized RNA was added in equal quantities to each sample (spiked in controls) for data normalization, as described elsewhere [44], to allow the comparison of samples from different growth stages on the same microarray.

**Figure 2:**
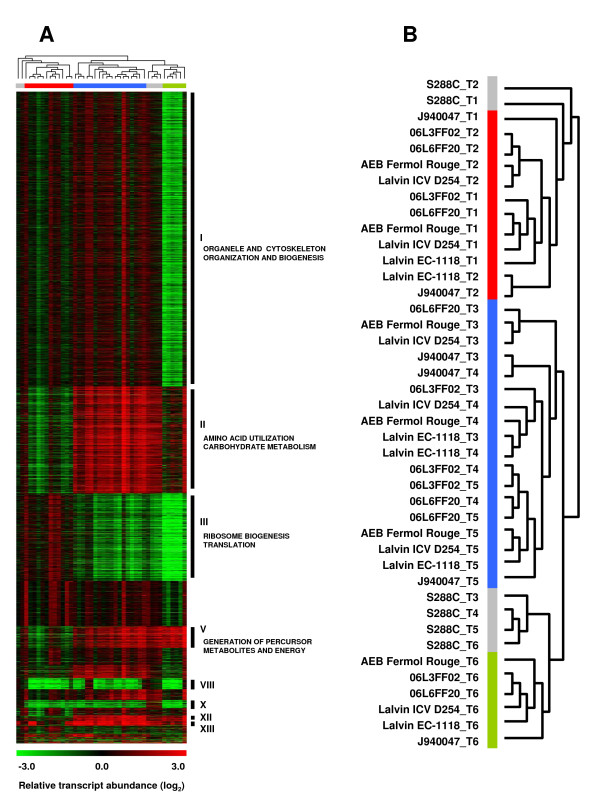
**Transcriptome profiles of yeast strains**. A) Hierarchical clustering (Pearson correlation) of the relative transcript abundance profiles (log_2 _scale) of the yeast strains at different fermentation stages (see text for details) indicates similarities and differences in gene expression regulation along the fermentation process. B) Gene clusters highlight differences in the transcriptome profiles of cells in exponential growth (T1 and T2) and in stationary phase (T3 -T6). The gene expression profiles of strain S288C (Grey bar) were distinct form those of the other strains in all growth stages. The transcriptome profiles of strain J940047 were similar to those of the environmental and commercial strains but constituted, nevertheless, a distinct branch in the sub-groups defined by exponential growth profiles (Red bar), early stationary phase profiles (Blue bar) and late stationary phase profiles (Green bar). Thirteen groups of highly correlated gene co-expression patterns were identified by hierarchical clustering (see results section) and some of these clusters are highlighted in Panel A. Some of the enriched GO terms are indicated next to each cluster.

Hierarchical clustering of the relative transcript abundance profiles revealed common trends in global gene expression at each of the fermentation stages (Figure 2). Profiles from exponential growth time points (T1 and T2) were very distinct from those obtained at later fermentation stages, irrespective of the strain (Figure 2B). The profiles obtained for the clinical J940047 and the laboratorial S288C strains were distinct from those of the wine related strains throughout fermentation. In fact, transcriptional profiles of the clinical strain J940047 grouped at marginal positions from the clusters formed by winemaking strains at T1/T2, T3/T4/T5 and T6 while profiles obtained for strain S288C grouped apart in a single cluster corresponding to several fermentation time points (T3/T4/T5/T6) and two separate positions (T1 and T2). Transcriptional responses of the commercial strains AEB Fermol Rouge and Lalvin ICV D254 were very similar at time points T2, T3 and T5 and a similar tendency was observed for environmental strains isolated from the Bairrada region (06L3FF02 and 06L3FF20), which had similar profiles at time points T2 and T6. Strains Lalvin EC-1118 and J940047 had similar expression profiles at the metabolic transition and in early stationary phase (T3 and T4 time points, respectively) while strains 06L3FF02, 06L3FF20 and S288C had similar transcriptome profiles at T4 and T5 (Figure 2B). The separation of the profiles obtained at T6 for the environmental and commercial strains was due to the sharp decrease in relative transcript abundance of many genes in these strains.

Common gene expression trends were observed among the investigated strains. Growth arrest (T3) coincided with a strong induction of genes involved in glycolysis, thiamine biosynthesis and ergosterol uptake, together with genes coding for the PAU/TIR family of cell wall proteins, in agreement with previous reports [42,45,46]. At this stage, the fermentation stress response was induced and heat shock proteins (*HSP78, HSP26 *and *HSP30*), together with proteins involved in cell wall organization and biogenesis, glucose uptake, iron homeostasis and vacuolar polyamine transport, among others, were up-regulated [47]. The decreased expression of many genes involved in cell cycle progression and nutrient response was also common to all strains throughout culture progression [34-36].

### Differential expression of co-regulated genes

Hierarchical clustering of gene expression profiles across samples revealed 13 clusters with highly correlated gene expression patterns (Figure 2A). The averaged expression profiles of each cluster highlighted differences between strains (Additional file 2). Strains J940047 and S288C were distinguished from the environmental and commercial strains by the averaged profile of many of these clusters, particularly at the time point T6. These differences could be related to differences in the transcriptomic response due to slower fermentation rate, indicating deficient adaptation to these growth conditions. As a result of a fermentation delay, strains J940047 and S288C did not show the general repression in transcription at T6 which was characteristic of the environmental and commercial strains. Enrichment analysis of function and transcription factor binding sites of genes listed in each cluster pointed to cellular functions and metabolic pathways that distinguished the strains. The most relevant results are summarized in Table 1.

**Table 1:** Predominant functional annotations obtained for genes found in selected clusters from Figure 2

Cluster^1^	Functional categories^2^	TF^3^	Selection of genes
II	Carbohydrate metabolism Energy reserve metabolic processes Oxidative phosphorilation Utilization of amino acids as N source Glutamate pool management Amino acid transport	STRE element Msn2p Msn4p Adr1p Sut1p	*DAL2-4, DAL82, DUR1,2, PUT2, GDH2, GDH3, GAP1, UGA4*

V	Generation of precursor metabolites and energy Trans-membrane transport Pleiotropic drug resistance Amino acid biosynthesis and transport	Adr1p	*SNF1, JEN1, CAT8, ADR1, MAL31, HXT5, AZR1, PDR10, YOR1, CRS5, TPO2, TPO3, MUP3, STR3, MET32, PUT4*

X	Sugar transport Pheromone signaling Mating Meiotic recombination	No enrichment	*MAL11, MPH2, BAR1, STE2, STE6, PRM8, ASG7, FUS1, GPA1, MFA2, STE18, HO, REC102, SPO11*

XII	Response to toxins Nitrogen catabolite repression	No enrichment	*AAD4, AAD6, AAD14, AAD16, ADH7, DAL80*

XIII	Sterol metabolism Thiamine biosynthesis Cell wall composition Iron homeostasis Pleiotropic drug resistance	Mot3p Nrg1p Aft2p Cin5p	*DAN1-DAN4, TIR4, PAU1-PAU6, ARE1, HES1, FET4, PDR11*

^1^Clusters highlighted in Figure 2A; ^2^Derived from GO term enrichment analysis (*p*-value < 0.05); ^3^Obtained from enrichment analysis for transcription factor binding site in the gene promoter region (*p*-value < 0.05, adjusted with Bonferroni multiple tests correction).

The expression of 2891 genes grouped in cluster I (Figure 2A) corresponded to increased biomass production. The main functional categories of the genes included in this cluster were RNA processing, vesicle-mediated transport, and organization/biogenesis of organelles and cytoskeleton. No statistically significant enrichment for transcription factor binding sites was found due to the wide representation of cellular functions in this dataset. On other hand, many of the genes grouped in cluster II (Figure 2A) were annotated to amino acid utilization, carbohydrate metabolism, oxidative phosphorylation and other processes related to energy reserve metabolism. This cluster was enriched in genes whose expression is controlled by Msn2p and Msn4p transcription factors through the STRE cis-elements present in stress genes [13,14], including fermentation stress response genes [47]. The transcriptional response to changes in carbon source utilization and hypoxia was also represented in cluster II, since it was enriched in genes with binding sites for Adr1p and Sut1p transcription factors (Table 1). These genes were repressed at T6 in the environmental and commercial strains but strains J940047 and S288C maintained the expression levels reached in the T4-T5 time points.

The expression profile of genes represented in cluster III (Figure 2A) decreased gradually throughout fermentation in all strains. These genes are regulated by the transcription factors Sfp1p, Rap1p and Azf1p which control the expression of protein synthesis factors. Many of the cluster III genes are also annotated to cell cycle progression and response to nutrients and had similar regulation in all strains. However, *MUP1 *and *CYS3 *(involved in sulphur amino acid transport and metabolism) showed a different behaviour from the general pattern of decreased expression, as 06L6FF20, Lalvin ICV D254 and J940047 strains up-regulated these genes at T3 and T4 and down-regulated them at T5.

Cluster V (Figure 2A) included genes involved in the transition from fermentative to oxidative metabolism, and many of them are regulated by Adr1p, a transcription factor responsible for induction of glucose repressed genes [48]. Removal of glucose repression was supported by the expression profiles of *SNF1 *which is required for transcription of glucose-repressed genes, *JEN1 *which is a lactate transporter gene repressed in the presence of glucose, and *CAT8 *which codes for a transcription factor that regulates the expression of a variety of genes under non-fermentative growth conditions. Other genes included in cluster V are involved in sugar transport (*MAL31 *and *HXT5*), multidrug resistance (*AZR1, PDR10, YOR1 *and *CRS5*), methionine transport and biosynthesis (*MUP3, STR3 *and *MET32*) and proline transport (*PUT4*) (Table 1). This is in agreement with previous studies showing that methionine and proline are important for tolerance to different stress factors [49-51]. All strains maintained high expression levels of these genes from T3 throughout T6. Relatively elevated transcription levels were observed in strain J940047 at T5 and T6.

**Figure 3:**
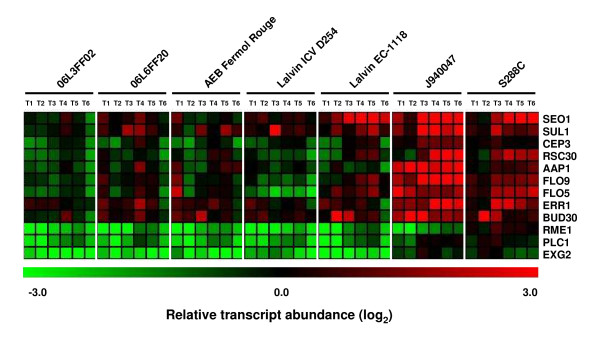
**Gene expression variability distinguishes yeast strains**. Expression of genes involved in flocculation, transport and cell division distinguished strains J940047 and S288C from the environmental and commercial strains. When compared to the environmental strains (06L3FF02, 06L6FF20) and the commercial strains (AEB Fermol Rouge, Lalvin ICV D254 and Lalvin EC-1118), strains J940047 and S288C showed higher relative transcript abundance for genes included in cluster VIII of Figure 2A throughout fermentation. Among these were Ty, flocculation (*FLO5 *and *FLO9*), transport (*SEO1 *and *SUL1*), cell wall organization and biogenesis (*EXG2*) and meiosis (*RME1 *and *CEP3*) genes. All strains up-regulated these genes at the transition from exponential to stationary phase, but the increase in expression was more pronounced in the case of strains J940047 and S288C. Only the genes with annotated functions are depicted in the heat map, for comparison.

Environmental and commercial strains differed markedly from J940047 and S288C in the expression of genes belonging to cluster VIII. Genes involved in flocculation, cell wall organization/biogenesis, transport and cell division (Figure 3), together with many Ty element ORFs, showed higher expression levels in strains J940047 and S288C relative to the environmental and commercial strains regardless of the fermentation time point. In a previous study [37], we identified gene copy number variation in the genomes of these strains which included some of the genes present in cluster VIII, namely *SEO1 *and *RSC30 *genes (see Additional file 3 for details on relative genome hybridization pattern). Meanwhile, cluster X grouped the expression profiles of genes that could be used to distinguish strain S288C from the other strains throughout the fermentation process. Most of the 79 genes of this cluster corresponded to non annotated ORFs while annotated ORFs were linked to sexual reproduction (Table 1), Ty elements and included the *ASP3 *tandem genes which are copy number depleted in the other strains relatively to strain S288C [37].

Genes included in clusters XII and XIII were highly expressed in all strains during early stationary phase (T4 and T5) (Figure 2A). Many of these genes are involved in stress resistance and are frequently located (~45%) in sub-telomeric regions [52]. The negative regulator of the nitrogen catabolite repression *DAL80 *was found in this cluster (Table 1) and its expression was most variable at T3 among the strains (results not shown). Many of the genes included in cluster XIII are responsive to anaerobiosis and were enriched in Mot3p and Nrg1p transcription factor binding sites (Table 1). Genes required for iron homeostasis and pleiotropic drug resistance were also represented in this cluster, as were genes with promoter binding sites for Aft2p and Cin5p, which regulate resistance to oxidative stress (Table 1).

Still noteworthy was the behavior of a group of 36 genes that shared a common trend in expression regulation, although with a relatively low correlation coefficient (< 0.657). Many of these genes were *MET *genes located in sub-telomeric regions. Their relative expression peaked in early stationary phase in all strains but was higher, on average, in strain J940047 when compared to the other strains.

### Differential gene expression among yeast strains

**Figure 4:**
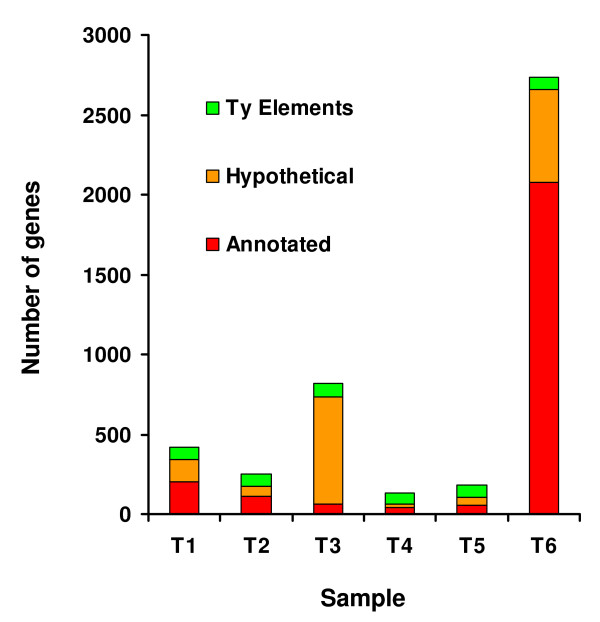
**The higher expression levels of many genes distinguishes S288C and J940047 from the environmental strains**. The higher expression of many genes was characteristic of strains J940047 and S288C when compared to the environmental (06L3FF02, 06L6FF20) and commercial (AEB Fermol Rouge, Lalvin ICV D254 and Lalvin EC-1118) strains. These differences were observed throughout fermentation, being particularly evident at the end (T6), and impacted stress response genes, as well as genes annotated to metabolic pathways relevant for fermentation progression. Many of the differentially expressed genes were hypothetical or corresponded to Ty elements, as is indicated by colour coded bars.

The global transcriptome profiling data (Figure 2) showed that the gene expression profiles of strains J940047 and S288C were distinct from the others. To further characterize such differences, a significance analysis (SAM) was performed, considering strains J940047 and S288C as a group and the environmental and commercial strains as another group. Strains J940047 and S288C had relatively higher expression of many genes (Figure 4), which was consistent throughout fermentation, and this trend affected in particular annotated ORFs at T6 and non-annotated ORFs at T3. Ty element ORFs were differentially expressed throughout fermentation.

Enrichment of transcription factor binding sites of the differentially expressed ORFs was used to identify the main functional differences between the two groups of strains at each fermentation stage. At T1, genes involved in the stress response, DNA damage and hypoxia showed lower expression in the environmental and commercial strains, as indicated by the enrichment of this dataset for genes with binding sites for Msn2p/Msn4p, Rph1p and Sut1p transcription factors, respectively. The same strains showed, in general, lower expression of genes required for the utilization of poor nitrogen sources, ethanol, glycerol and fatty acids, as supported by the enrichment of the same dataset for promoter regions responsive to Adr1p and Dal82p. The same was observed for genes controlled by Mcm1p, (regulation of pheromone response). At T2 (exponential growth), strains J940047 and S288C had higher expression of genes involved in thiamin and vitamin B6 biosynthesis. This pattern was also observed for several genes involved in oxidative phosphorylation and in the homeostasis of TCA cycle precursors, such as succinate (*SDH2 *and *SDH3*) and NAD (*BNA1, BNA5*, and *TNA1*).

In stationary phase, in particular at T4, strains J940047 and S288C displayed higher expression of genes controlled by the transcriptional regulators of methionine biosynthesis Cbf1p and Met31p. Concomitantly, many genes involved in sulphur amino acid biosynthesis [53] also had higher expression intensity in these strains. At T5, no functional enrichment was found for the group of differentially expressed genes, while genes with higher expression in strains J940047 and S288C at T6 were enriched in promoter binding sites for Rap1p and Sfp1p, which regulate ribosome biogenesis in response to nutrients and stress. This general transcription repression involved ~2000 genes and constituted a shared regulatory response of environmental and commercial strains to the changed environmental conditions at the end of fermentation.

**Figure 5:**
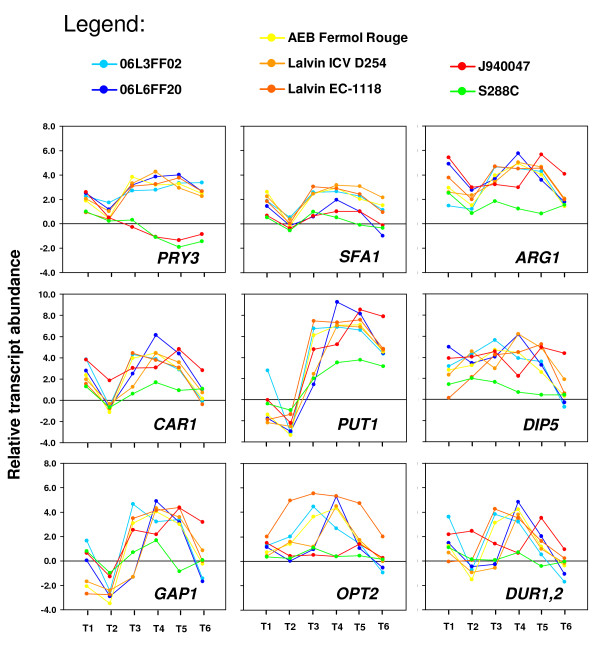
**Response to nitrogen depletion differentiates winemaking strains**. Differential expression analysis revealed that 14 genes at T4 had higher expression levels in the environmental (06L3FF02, 06L6FF20) and commercial (AEB Fermol Rouge, Lalvin ICV D254, and Lalvin EC1118) strains than in J940047 and S288C strains. Most of these genes are required for utilization of poor nitrogen sources, indicating differences between the strains in the response to nitrogen depletion. The panels depict the relative transcript abundance of some of these genes in each strain.

SAM analysis identified a small group of 14 genes whose expression intensity was higher in environmental and commercial strains, in particular during early stationary phase (T4). Expression of *PRY3 *genes, encoding a cell wall homologue of plant PR-1 proteins was strongly induced in these strains and was repressed in strains J940047 and S288C from T3 to T6 (Figure 5). The majority of the genes belonging to this group are involved in alternative nitrogen source utilization. For example, *DIP5 *and *GAP1 *genes encode amino acid transporters, and *SFA1, ARG1, CAR1 *and *PUT1 *genes are involved in amino acid metabolism (Figure 5). The plasma membrane oligopeptide transporter gene *OPT2 *also showed expression variability between the groups of environmental and commercial strains. Strain Lalvin EC-1118 showed a strong expression of *OPT2 *at T2 while other strains achieved maximum expression values during late fermentation stages (T3 or T4). Interestingly, environmental and commercial strains could also be distinguished by differential expression of allantoin and urea catabolism genes, namely *DUR1,2 *(Figure 5), *DAL7 *and *DUR3 *(not shown).

### Major sources of gene expression variability

**Figure 6:**
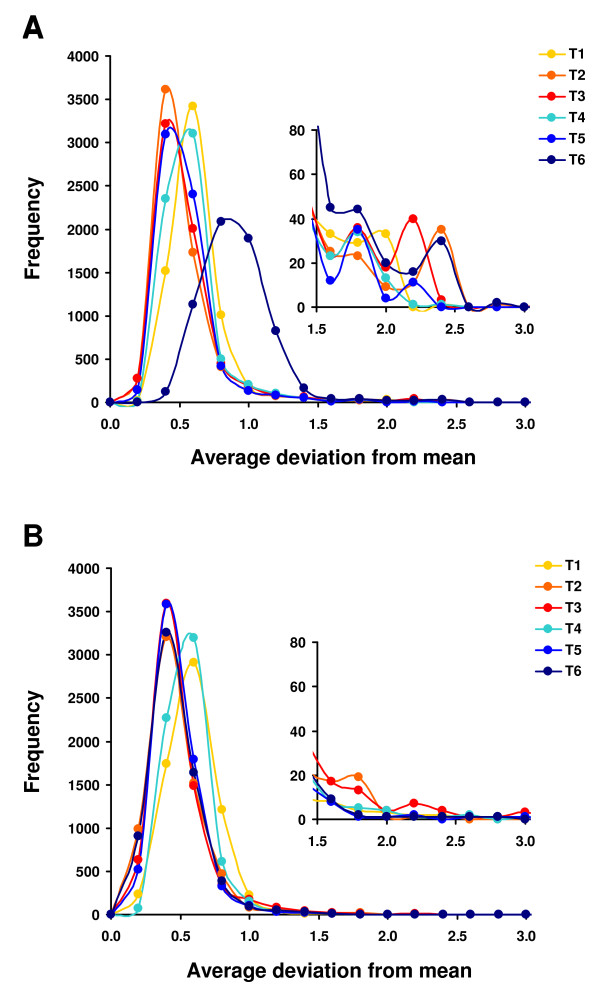
**Variability in gene expression is enhanced at the beginning of fermentation and during early stationary phase**. Variability in gene expression was compared from T1 to T6 fermentation time points, considering the average deviation from the mean expression value as a measure of amplitude of gene expression. Panel A shows the distribution of the average deviation values for all strains and Panel B represents the same distribution for the environmental (06L3FF02, 06L6FF20) and commercial (AEB Fermol Rouge, Lalvin ICV D254 and Lalvin EC-1118) strains only. Inserts in Panels A and B expand the scale to highlight the frequency of highly variable genes.

Gene expression differences between the yeast strains were investigated at the monitored growth stages (Figure 6). The highest amplitudes of gene expression were registered at T6 (Figure 6A), but this was a reflection of the extensive transcriptional repression that occurred in environmental and commercial strains at this stage of fermentation (Figure 6B). Variability was biased towards TATA box genes (Additional file 4), in agreement with what was reported by Basehoar and colleagues [54], who showed that TATA box containing genes comprise approximately 20% of the yeast ORFeome and are highly regulated in response to stress. Our results reinforce the hypothesis that this promoter element is an important generator of expression diversity under environmental pressure.

**Figure 7:**
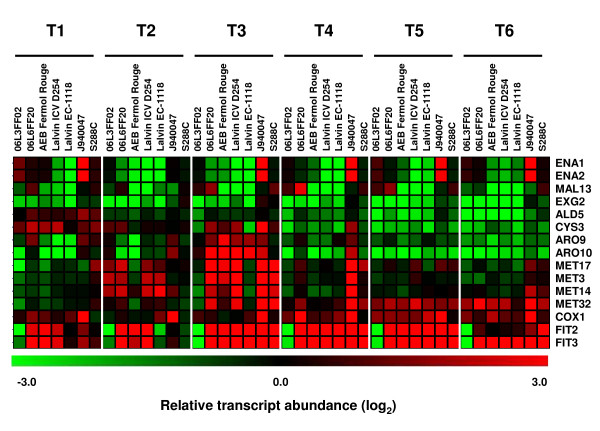
**The expression of aromatic compound synthesis genes is highly variable**. Highly variable genes were identified at several fermentation stages, with particular incidence for genes required for the production of fusel alcohols and other aromatic compounds. Examples of this were *ARO9 *and *ARO10 *genes whose products are part of the Ehrlich pathway, *CYS3 *and several *MET *genes involved in the production of sulphur containing volatile compounds and *EXG2*, which is required for the production of volatile glycosides. Their relative transcript levels and those of other highly variable genes is shown.

Among the genes with highest expression variability, namely those with an average deviation from the mean of the relative transcript abundance above 1.5 (see inserts in graphs of Figure 6A and 6B), were many genes whose transcription is relevant for fermentation progress (Figure 7). For example, *ARO9 *and *ARO10 *genes that code for proteins involved in the metabolism of aromatic amino acids and production of fusel oils via the Ehrlich pathway, showed the highest expression variability at T1 and T2. Also *CYS3 *and several *MET *genes, whose products are involved in the production of sulphur containing volatile compounds, were most variable at T3. The expression of *ALD5 *(conversion of pyruvate to acetate) and *EXG2 *(production of volatile glycosides) distinguished the yeast strains at T6. Expression of *ENA1 *and *ENA2 *(involved in the response to osmotic stress) was highly variable irrespective of the fermentation stage and this variability was due to their elevated expression in strain J940047 relative to the other strains. The expression of *MAL13 *(maltose fermentation) was divergent during stationary phase among the environmental and commercial strains. The transcription levels of *COX1, FIT2 *and its homologue *FIT3*, were most variable from T2 to T6, suggesting differences in redox homeostasis among the yeast strains from early stages of fermentation. The *COX1 *gene showed the highest relative transcript abundance in strain J940047 and the *FIT2 *and *FIT3 *homologues had lower expression in strain 06L3FF02.

The cellular functions affected by gene expression variability were identified by functional enrichment analysis performed on a ranked list of genes [55], considering the average deviation from the mean of the relative transcript abundance calculated for each gene as the ranking criterion. Genes involved in transposition were highly variable during all fermentation time points, most probably due to the high variability in Ty copy number among yeast strains [37,56,57]. Genes annotated to disaccharide and pyridoxine metabolism, vitamin B6 biosynthesis and aspartate family amino acid metabolic process also had highly variable transcript abundance throughout fermentation.

Top variance in gene expression was associated with the specific metabolic requirements of the corresponding growth phase (Additional file 5). For instance, genes involved in rRNA modification and processing were the most variable at T1, while genes annotated to mitochondrial transport and to metabolism of intermediate metabolites of the TCA cycle were the most variable at T2. Both T1 and T2 stages had high variability in the expression of genes involved in oxidative phosphorylation, transport of ions and glucose, and in regulation of nucleotide metabolism. In particular, T3 revealed high expression variability of genes involved in the cycloheximide and fatty acid metabolism. Expression of genes involved in thiamine and steroid biosynthesis, together with genes related to amino acid metabolism and transport, differentiated the yeast strains during stationary phase.

In order to identify possible sources of gene expression variability, the transcript profiling data was cross-checked with comparative genome on array (aCGH) data, obtained in our laboratory in a previous study [37]. Among the genes that were copy number depleted in the environmental and commercial strains relatively to strains J940047 and S288C (Additional file 3) were the top variable genes *FLO5, FLO9, PLC1, RSC30 *and *SEO1*. The relative transcript abundance of these genes was also comparatively lower in the environmental and commercial strains when compared to strains J940047 and S288C (Figure 3). We further investigated the correlation between gene expression and gene load in the T2 and T4 fermentation time points by calculating the relative transcript abundance for every strain relatively to that of strain S288C. Several correlations (Additional file 6) were dependent on the fermentative time point (T2: *MAL13 *and *MAL33*; T4: *ENA5, PLC1, SEC23 *and *SPT7*). A correlation between gene load and gene expression both at T2 and T4 was identified only for *AGC1 *and *HSF1*. Interestingly, these genes, together with *SPT7 *and *SEC23*, were amplified in strain J940047 relative to the other strains (Additional file 3).

## Discussion

### Differences in fermentation profiles among yeast strains

This comparative transcriptomics study of *S. cerevisiae *strains isolated from Portuguese vineyards, (06L3FF02 and 06L6FF20), commercial wine strains (AEB Fermol Rouge, Lalvin EC-1118 and Lalvin ICV D254), a clinical isolate (J940047) and the laboratory strain S288C showed important mRNA content variability among them. All strains were able to complete fermentation and yielded similar biomass and ~ 10% (p/v) of ethanol concentration. However, winemaking strains had short latency time and completed fermentation earlier (~ 4 days). No differences in growth profile were observed between the group of commercial strains and environmental strains isolated from vineyards. This is in agreement with recent data showing that winemaking strains form a homogeneous group with similar behaviour under winemaking conditions, irrespective of their geographical origin [7-9]. Nevertheless, several studies showed significant phenotypic variation among winemaking strains [30,58] and, in spite of the similarity in fermentation profile, the commercial strains Lalvin ICV D254 and AEB Fermol Rouge could be differentiated from the other wine related strains on the basis of their transcriptomic profiles (Figure 2B) throughout fermentation (T2, T3 and T5).

The clinical (J940047) and laboratorial (S288C) strains fermented slower than the wine making strains. The global hierarchical clustering of transcriptomic profiles obtained for all strains and time points confirmed that strain S288C was more dissimilar, while the clinical strain J940047 was more similar to wine related strains (Figure 2B), suggesting that the latter may have originated from wine strains. A recent analysis of genome-wide patterns of nucleotide polymorphisms showed that clinical strains do not derive from a common ancestor and that European clinical strains are closely related to wine strains, which is in agreement with our data [7,8]. Conversely, strain S288C was obtained in the 1950's through genetic crosses and 88% of its gene pool originated from strain EM93 which was initially isolated from a rotten fig [59]. Therefore, the cultivation of strain S288C over decades under laboratory conditions may have altered its fermentative performance and ethanol resistance. This is supported by a recent study showing that S288C is particularly sensitive to ethanol when compared to other 52 *S. cerevisiae *strains isolated from a variety of biotopes [30].

### The transition from fermentation to respiration differentiated yeast strains

The entry into stationary phase was accompanied by increased transcription of numerous genes (clusters II and V of Figure 2), coinciding with the shift from fermentative to oxidative metabolism [60]. Among the genes whose expression was reprogrammed were many genes that are controlled by the transcription factor Adr1p, which regulates genes that are under glucose catabolite repression [61]. The relief of glucose catabolite repression under similar conditions of high sugar concentrations has been linked to nitrogen depletion which occurs during must fermentation [42,62]. The ability to ferment and respire simultaneously is known as the Crabtree effect and is characteristic of the yeast species that underwent whole-genome duplication [63]. However, environmental and commercial strains showed smaller amplitude in the up-regulation of oxidative phosphorylation and TCA cycle genes at the metabolic shift (stage T3) relative to the other strains and this cannot be solely attributed to differences in glucose concentration during the respective fermentations as the metabolic flux partitioning between fermentative and oxidative metabolism is associated with the demand for NADPH rather than to the need of carbon precursors [64]. Respiratory metabolism, together with the fermentative stress response, was more up-regulated in strains J940047 and S288C than in the environmental and commercial strains, highlighting the importance of anaplerotic reactions for the cellular management of multiple stress factors.

### Expression of nitrogen metabolism genes was highly variable

Nitrogen metabolism reorientation occurs upon depletion of assimilable nitrogen and was observed at stages T4-T6, corresponding to growth arrest. The amplitude and the timing of gene expression related to nitrogen catabolite repression were particularly diverse among the environmental and commercial strains. *CAR1 *gene (coding for an arginase) and *PUT1 *gene (whose product is a proline oxidase) exemplify this trend (Figure 5), among many others included as part of clusters II and XII (Figure 2). The tight regulation of these genes is of paramount importance for the fermentation progress and outcome [45], in particular if one considers that arginine and proline are the most abundant nitrogen sources in grapes [65]. On another hand, strain S288C had the lowest expression levels for *PUT1*, as proline was among the least-preferred nitrogen sources in strains with S288C background [66]. The different utilization kinetics of these and other amino acids are tightly linked with the sensorial properties of wine because amino acids are important precursors of volatile compounds [67,68]. Therefore, differences in the expression of these genes in winemaking strains throughout fermentation may be one of the reasons for divergent aroma profiles of wines obtained with distinct *S. cerevisiae *strains.

The expression of *OPT2 *(Figure 5), encoding a membrane peptide permease [69], was strongly up-regulated in stationary phase in the environmental and commercial strains. Each wine strain showed a characteristic expression profile of *OPT2 *in both timing and amplitude of expression. This indicates important variability in the capacity of strains to survive in environmental conditions which require the utilization of small peptides as nutrients [70]. A similar situation was observed for *DUR1,2 *(Figure 5), which indicates considerable inter-strain variation in urea degradation to CO_2 _and NH_3 _as an additional resource for assimilable nitrogen. In fact, differences in the transcriptional level of regulators of nitrogen catabolite repression such as *DAL80 *and *GAT1 *were registered during growth arrest (T3), although without statistical significance. The reciprocal negative feedback mechanism controlled by those two genes [71] regulates the expression of amino acid and ammonium permeases and of some proteases involved in protein degradation. The high relative expression levels of those regulators towards the end of fermentation in strain J940047 (results not shown) highlights an enhanced response to long term depletion of assimilable nitrogen. Diversity in the expression of amino acid permeases and in oligopeptide uptake via OPT2 were previously identified among natural isolates of wine yeast from Tuscan vineyards [11].

Particularly interesting was the higher relative expression of *SFA1 *in the winemaking strains, both from environmental and commercial origin, throughout stationary phase (Figure 5). In yeast, glutathione-dependent formaldehyde dehydrogenase activity of the protein encoded by this gene plays an important role in the last step of fusel alcohol production via the Ehrlich pathway [72]. While these compounds are responsible for flavour and aroma of yeast-fermented foods and beverages, they are also involved in the induction of filamentous growth in *S. cerevisiae *and biofilm formation in the pathogens *Candida albicans *and *Candida dubliniensis *[72]. In the environmental and commercial strains, the expression profile of *SFA1 *was similar to that of *PRY3 *(Figure 5) which is involved in cell wall biogenesis [73]. Therefore, the expression levels of these two genes may be linked since Pry3p expression is up-regulated in response to organic solvents [74].

### The origin of gene expression diversity

Yeasts have the ability to regulate gene expression when challenged by pleiotropic stress conditions, such as those imposed by fermentation of high sugar and low nitrogen substrates. Gene expression divergence among closely related strains in response to stress may be due to genomic diversity [25,75], namely by the accumulation of polymorphisms in *cis- *and *trans*-acting regulatory factors [76,77], together with epigenetic factors [78]. Small variations in gene content, even among yeast strains with close phylogeny [30], may also alter gene expression when regulatory genes are affected, while the expression of genes of exogenous origin [79] may even confer or complement phenotypes that allow for survival under specific stressful environments.

The expression of genes involved in various aspects of nitrogen and amino acid metabolism was highly variable among the studied strains. Variability in expression of amino acid metabolism genes supports previous comparative transcriptome analysis of yeast isolated from a wider range of biotopes [10,11,30], but the mechanisms responsible for gene expression variability among phylogeneticaly related strains is not understood. Genome polymorphisms [26] explain part of that variability, however many environmental stress responsive genes, namely amino acid metabolism genes [14], may be under "noisy" expression control. Indeed, stress response genes often possess TATA boxes in their promoter regions [54] and the sensitivity of gene expression to mutations increases both with increasing trans-mutational target size and the presence of the TATA box [80]. The TATA box is associated with high gene expression variance in yeast and many other organisms [81], and our study showed that gene expression variability was correlated with the TATA box in a strain independent manner, supporting previous findings [30]. This association between the TATA box and gene expression variability has been attributed to a higher rate of regulatory divergence for genes with this cis-element, either in response to selection [81] or mutation accumulation [80]. But, genes with the TATA box show high variability in expression even in cultures of the same strain [77,82] grown in different media conditions, supporting that the expression of these genes is also responsive to environmental or genetic perturbation [30]. In other words, the TATA box is a potential generator of phenotypic diversity under environmental selection within an isogenic population.

Retrotransposon activity is another mechanism by which phenotypes can evolve in yeast. Ty element mobility has been associated to genome divergence between yeast strains and species [76,83] and insertion of these elements result frequently in chromosomal rearrangements and gene duplications [38]. The relative abundance of Ty elements in the genome is related to the genetic background of the strains [37,56,57] and yeast strains differ in their relative expression. Previous studies [12,30] differentiated strain S288C from other strains relatively to expression of Ty genes, showing good correlation between expression intensity and relative Ty copy number [30]. Our data support that correlation, since Ty transcription levels, which were higher in S288C and J940047 strains relative to commercial and environmental isolates throughout fermentation, were correlated to increased Ty abundance [37]. Interestingly, the higher expression of Ty elements coincided with higher levels of expression of stress responsive genes in strains S288C and J940047, raising the hypothesis of a cause-effect relationship between these two variables.

Positive correlations between gene expression level and gene load were only found for few genes with annotated functions (Supplementary Figure S5). However, differences in the expression of some of these genes may have a direct impact in the transcription levels of many others, thus contributing to the general variability in expression observed among the investigated strains. For example, expression differences in *HSF1, PLC1 *and *SEC23 *were correlated with putative gene load differences (Additional file 6); the products of these genes have direct impact on many cellular processes. Hsf1p is a transcription factor that controls the expression of hundreds of genes involved in protein folding, detoxification, energy generation, carbohydrate metabolism and cell wall organization [84] and is part of a global molecular response to diverse stress stimuli linked to protein misfolding [85]. The *PLC1 *gene product regulates nutrient sensing, filamentous growth, PKA-mediated stress response [86,87], cellular processes that may have a direct impact on yeast pathogenicity [88,89], while *SEC23 *plays a role in the yeast secretory pathway [90] and influences protein secretion and the protein pool at the yeast-environment interface. Copy number variations in *PLC1 *and *SEC23 *may be common among yeast strains, since both are sub-telomeric and are, therefore, subjected to the instability of these genomic regions [40]. Also interesting was the *SPT7*gene whose expression levels were higher in strains with putatively increased gene load. The product of this gene is a subunit of the SAGA complex which is a transcription activator of RNA polymerase II-dependent genes [91]. The Spt7p load determines the stoichiometry of the SAGA complex due to its involvement in proper complex assembly and control of other core subunits [92]. Since transcription activation of TATA box-containing genes occurs preferentially by the SAGA complex rather than TFIID [54], transcription of this gene should affect a huge variety of cellular functions due to broad functional distribution of the TATA promoter element.

## Conclusions

The present study shows that gene expression is variable among wild-type yeast strains. Such variability is observed throughout the spectrum of metabolic changes endured by yeast during glucose fermentation. The variability in expression levels of many genes impacted key aspects of yeast metabolism and can be seen as a potential generator of phenotypic diversity in yeast populations. Differences in gene expression during fermentation affected co-regulated genes and distinguished yeast strains. This indicates that gene expression changes in response to environmental challenges are not only a function of the intensity of the challenge, but are also determined by the genetic background of the strain. Therefore, a wider characterization of the variability of cellular responses and its connection to genomic traits is necessary to understand the plasticity and adaptability potential of natural yeast populations.

Differences between strains were enhanced at the beginning of fermentation, where the wine making strains had lower expression of stress- regulated genes, and during early stationary phase, when the expression of genes involved in the utilization of poor nitrogen sources was higher in the wine making strains. Interestingly, the clinical and the laboratorial strains showed higher expression of many non-annotated ORFs when ethanol production reached its maximum rate (T3). These observations suggest that winemaking strains cope better with stress-imposing environmental conditions and are able to manage limiting nutrients, namely nitrogen, in a more efficient and resourceful way. The regulation of expression of genes involved in nitrogen metabolism was one of the major sources of the diversity encountered among the winemaking strains while gene load differences, particularly those affecting key regulator genes, such as *HSF1 *and *SPT7*, were an important source of the diversity among all strains.

## Methods

### Yeast strains and culture conditions

Environmental strains were isolated from vineyards of the Bairrada wine region, Portugal, while commercial wine strains were kindly provided by Adega Cooperativa da Bairrada, Cantanhede-Portugal. The clinical isolate was a kind gift of Prof. Mick Tuite from the University of Kent, Canterbury, UK. Strain S288C is maintained as part of our laboratory's yeast culture collection. Details of the strains used in this study can be found elsewhere [37].

Fermentations were carried out at 24°C, using the culture medium MS300, commonly used as synthetic grape must [93]. Semi-anaerobic fermentations were carried out in 500 ml Erlenmeyer flasks filled with 550 ml of culture media and fitted with a Teflon stopper pierced with a syringe needle for gas exchange. Fermentations were inoculated to an initial OD_600 _of ~0.025 with the appropriate volume of overnight pre-cultures grown in the same culture medium. Homogenization of the cultures was carried out by magnetic stirring (500 rpm). CO_2 _production was determined by measuring culture weight loss during fermentation. The ethanol concentration was estimated from CO_2 _mass production using the equation E(g/l) = 1.011 mCO_2 _(g/l) + 2.7, as previously described [42]. Cells were harvested at the time points indicated in Figure 1 by centrifugation (3000 g, 3 min, at room temperature).

### RNA isolation and sample labelling

mRNA isolation, cDNA synthesis and labelling were carried out as described elsewhere [44]. Briefly, total RNA was isolated using hot phenol extraction. For hybridization quality control and normalization of the microarrays, mixtures of ten *in vitro *synthesised RNAs were added from appropriately diluted mixtures to 500 μg of total RNA in the case of T2 samples or to 1000 μg of total RNA in T1, T3, T4, T5 and T6 samples. Different amounts of total RNA were used for mRNA enrichment to account for differences in the relative amount of poly-A transcripts in total RNA extracts. mRNA enrichment of the samples was performed using Oligotex beads following the manufacturer's instructions for batch purifications (Qiagen). cDNA synthesis was carried out using 3 μg of mRNA enriched samples in the presence of 2-aminoallyl-dUTP. Samples were purified using Microcon-30 (Millipore) columns prior to coupling to Cy3 and Cy5 fluorofores. Before hybridization, free dyes were removed using Chromaspin-30 (Clontech) columns and the efficiency of cDNA synthesis and dye incorporation was measured by spectrophotometry (NanoDrop). Samples with a degree of labelling (labelled nucleotides per 100 nucleotides) outside the range of 5.0 ± 1.2 were not considered for hybridization.

### Microarray production

*In-house *spotted DNA-microarrays were prepared using 6388 oligonucleotide sequences (70 mer) targeting the complete ORFeome of *Saccharomyces cerevisiae *(OPERON Yeast AROS v1.1 collection, Qiagen). Probes were spotted twice on CodeLink activated slides (GE Healthcare) according to the slide manufacturer's instructions, using a MicroGrid Compact II spotter (GenomicSolutions) equipped with 48-quill pins (Microspot2500). A set of 70 mer probes, designed from *Escherichia coli *genome sequence with less than 70% homology to *S. cerevisiae *genome, was also included in the microarray. These probes were used to detect the spiked-in control RNA added to the total RNA sample in order to monitor labelling and hybridization quality. The array design and spotting protocol were deposited in ArrayExpress database under the accession code number A-MEXP-1185.

### Hybridization

Hybridizations were carried out as previously described [44], using a common reference design with dye-swap replicates. Total RNA obtained from strain S288C grown to mid exponential growth phase in MS300 medium was used as the reference sample. Four self-self hybridizations were performed using the common reference sample for control of experimental background. The raw data and the pre-processed data from a total of 88 hybridizations were submitted to the ArrayExpress Database and can be accessed using the code E-MTAB-112.

### Image acquisition and data processing

Images of the microarray hybridizations were acquired using the Agilent G2565AA microarray scanner and the fluorescence intensities were obtained with QuantArray v3.0 software (PerkinElmer). Pre-processing of the data was performed using the Biometric Research Branch (BRB)-ArrayTools v3.4.0 software. Manually flagged bad spots were eliminated and the local background was subtracted prior to averaging of replicate features on the array. Log_2 _intensity ratios (M values) were normalized using as reference the signal of five different control RNAs spiked in equal amounts to the samples co-hybridized in each array. The concentration of the spiked RNA controls was chosen so that log_2_(Cy5*Cy3)^½ ^) values (A values) would be distributed between 5 and 15. The log_2 _intensity ratios of the spiked-in controls were forced to a median value of zero and all data points were adjusted accordingly. Following data normalization, each pair of dye swap experiments was averaged to obtain the log_2 _intensity ratios representing the relative transcript abundance for each monitored ORF.

### Statistical analysis and functional annotation of the data

Hierarchical clustering of the normalised and dye-swap averaged samples was performed using Pearson correlation (average linkage) TM4 Microarray Software Suite (MeV) 4.3 [94]. Clustering of genes was carried out using CLICK, a clustering algorithm implemented in EXpression Analyzer and DisplayER (EXPANDER) 4.0.2 [95], which does not require prior assumptions on the structure or the number of clusters to be found. Thirteen highly correlated clusters of the expression profiles were obtained with an average homogeneity above 0,884.

Significance analysis was performed using Significance Analysis for Microarrays (SAM) [96] also implemented in MeV. Comparison of strains 06L3FF02, 06L6FF20, AEB Fermol Rouge, Lalvin ICV D254 and Lalvin EC-1118, against the non-wine related strains J940047 and S288C, at all fermentation stages tested was carried out with the two-class unpaired SAM test, allowing a maximum False Discovery Rate (FDR) of 8.7% (90% confidence), in the case of T4 samples. The same test was performed for the comparison of the laboratorial strain S288C against the other strains with a FDR (90% confidence) between 2.4% (T1 samples) and 1.5% (T6 samples).

The *Saccharomyces *Genome Database [55] was used for functional interpretation of the data. Enrichment of Gene Ontology term or transcription factor binding site was performed using TANGO and PRIMA algorithms, respectively, implemented in EXPANDER 4.0.2 software, using a *p*-value threshold of 0.05 (Bonferroni correction was used in PRIMA analysis). Gene set enrichment analysis on a ranked list of genes was performed using the FatiScan web tool implemented in Babelomics 3.1, using a two-tailed Fisher exact t-test with adjusted *p*-value cut-off of 0.05. The genes in the analyzed lists were ordered from the highest to the lowest average deviation from the mean of the relative transcript abundance value calculated across samples.

## Authors' contributions

LC printed the microarrays, performed the hybridizations, processed and analysed the data and wrote a draft of the manuscript. MFE helped with the hybridizations and data processing. ID performed the viability assays and helped with the hybridizations. DS participated in the design of the study, discussion of the results and the drafting of the manuscript. GRM corrected the manuscript. MASS coordinated the study and corrected the manuscript. All authors read and approved the final manuscript.

## Supplementary Material

Additional file 1 **Cell viability**. Cell viability during fermentation was compared for *Saccharomyces cerevisiae *strains Lalvin EC-1118, 06L3FF02, J940047 and S288C. Click here for file

Additional file 2 **Co-expression cluster line graphs**. Gene co-expression was investigated for strains 06L3FF02, 06L6FF20, AEB Fermol Rouge, Lalvin ICV D254, Lalvin EC-1118, J940047 and S288C from the measurements of relative transcript abundance during fermentation. Graphs represent the average relative transcript abundance (log_2 _ratio) determined for groups of genes having highly correlated expression profiles across samples. Click here for file

Additional file 3 **Relevant aCGH data**. Summary of gene copy number differences observed between the *Saccharomyces cerevisiae *strains included in this study. Relative gene copy number values (fold change relatively to strain S288C) were obtained by comparative genome hybridization on array (Carreto *et al. *2008. *BMC Genomics *9: 524). Only genes relevant for discussion in the present manuscript were represented. Click here for file

Additional file 4 **Variability in TATA box genes**. Variability in gene expression was biased towards TATA box genes. The graphics show the frequency of TATA box genes as a function of gene expression variability. The average deviation from the mean of the relative gene expression value was taken as a measure of expression variability. Panel A was obtained considering all the investigated strains while Panel B represents the distribution obtained for the environmental and commercial strains. Click here for file

Additional file 5 **GO Biological Process enrichment in top variable genes**. Results obtained from a Gene ontology (GO) term enrichment analysis performed to determine the over-representation of Biological Processes among the top variable genes (FatiScan). The list of genes was ranked according to the average deviation from the mean relative expression measured among the yeast strains studied. Click here for file

Additional file 6 **Correlation between gene expression and aCGH**. Correlation between relative transcript abundance and putative differences in gene load was found for some genes. The relative transcript abundance values determined for each strain relatively to those of strain S288C were plotted against the relative gene copy number differences for the same strains. The analysis was performed using the datasets from T2 (Panel A) and T4 (Panel B) stages of fermentation. Click here for file
