# Supplementary material for: Expression variability of co-regulated genes differentiates Saccharomyces cerevisiae strains
Source: BMC Genomics. 2011 Apr 20;12:201. doi: 10.1186/1471-2164-12-201 (PMC3094312; doi:10.1186/1471-2164-12-201)
Supplement: Additional file 1 — Cell viability. Cell viability during fermentation was compared for Saccharomyces cerevisiae strains Lalvin EC-1118, 06L3FF02, J940047 and S288C. [file 1471-2164-12-201-S1.PDF]

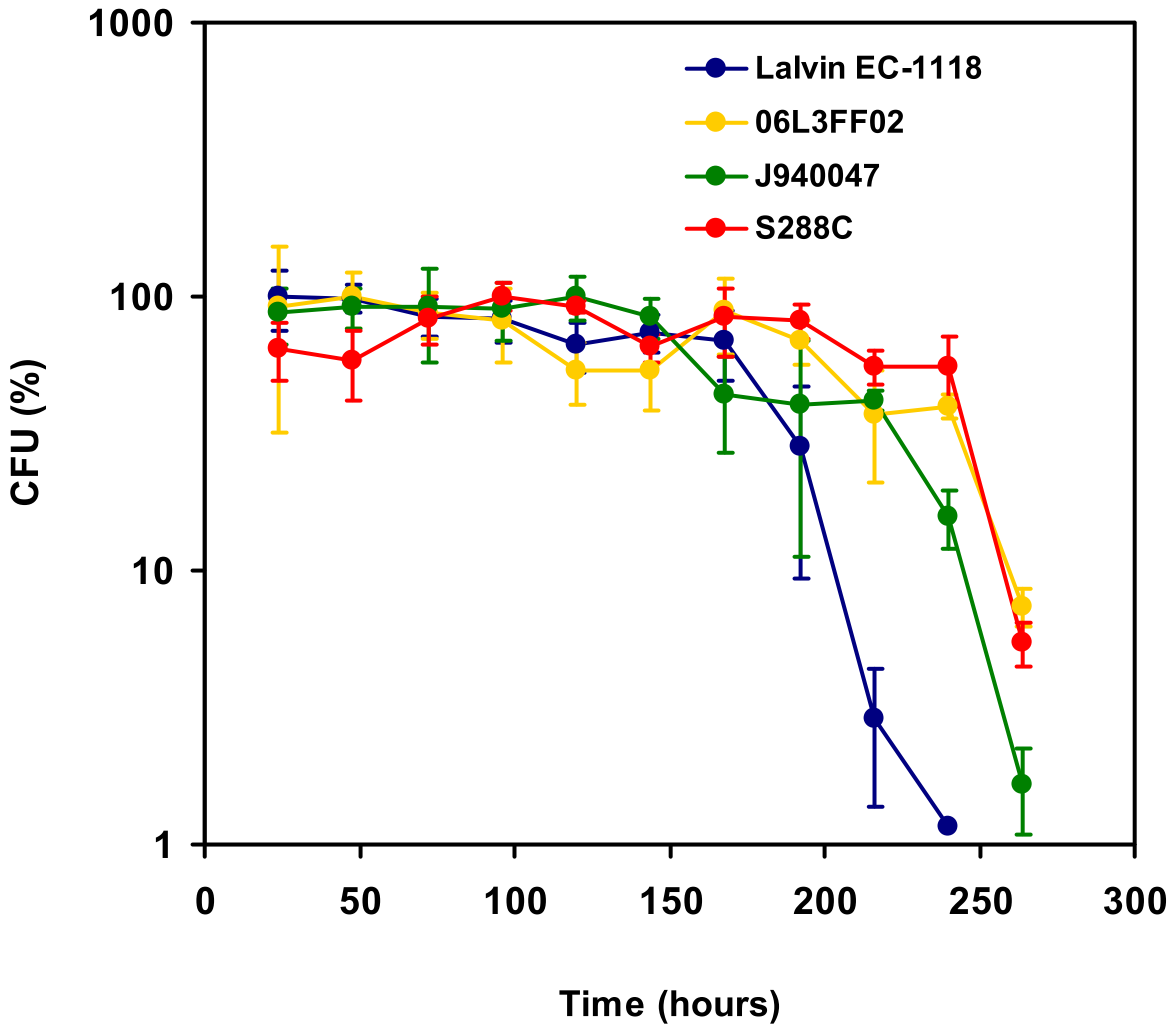

Supplemental Figure S1.
Evolution of cell viability during fermentation in synthetic wine must for *S. cerevisiae* strains Lalvin EC-1118, 06L3FF02, J940047 and S288C.
Colony forming units (CFU) were determined by plating serial diluted cell suspensions onto YPD agar plates. Plotted values (with standard error bars) correspond to the average CFU from triplicate plating of three independent cultivations.
