# Supplementary material for: Expression variability of co-regulated genes differentiates Saccharomyces cerevisiae strains
Source: BMC Genomics. 2011 Apr 20;12:201. doi: 10.1186/1471-2164-12-201 (PMC3094312; doi:10.1186/1471-2164-12-201)
Supplement: Additional file 2 — Co-expression cluster line graphs. Gene co-expression was investigated for strains 06L3FF02, 06L6FF20, AEB Fermol Rouge, Lalvin ICV D254, Lalvin EC-1118, J940047 and S288C from the measurements of relative transcript abundance during fermentation. Graphs represent the average relative transcript abundance (log2 ratio) determined for groups of genes having highly correlated expression profiles across samples. [file 1471-2164-12-201-S2.PDF]

Cluster I: 2891 genes

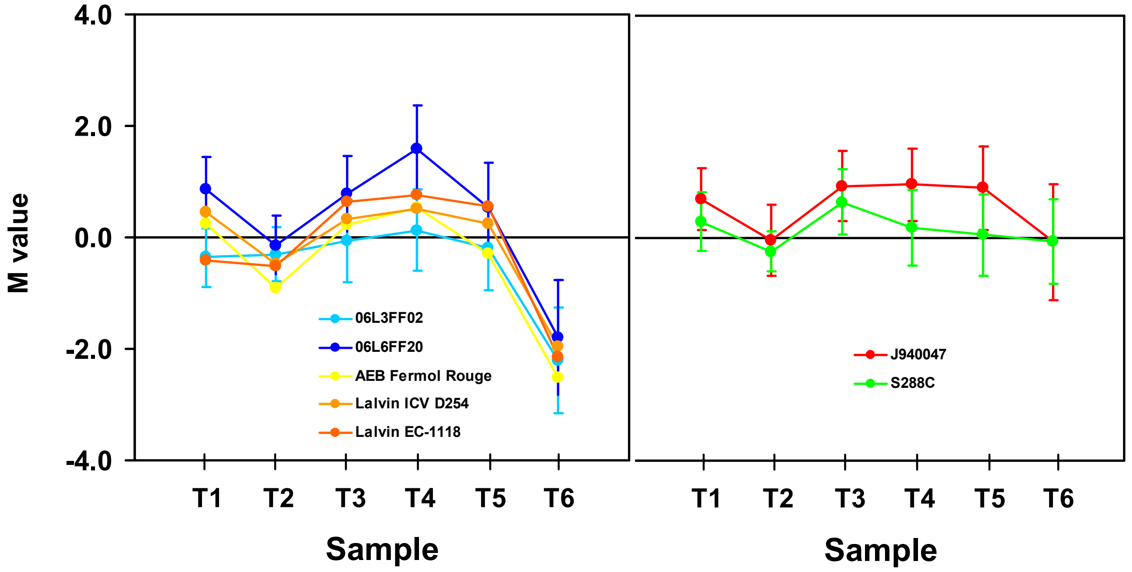

Cluster II: 1034 genes

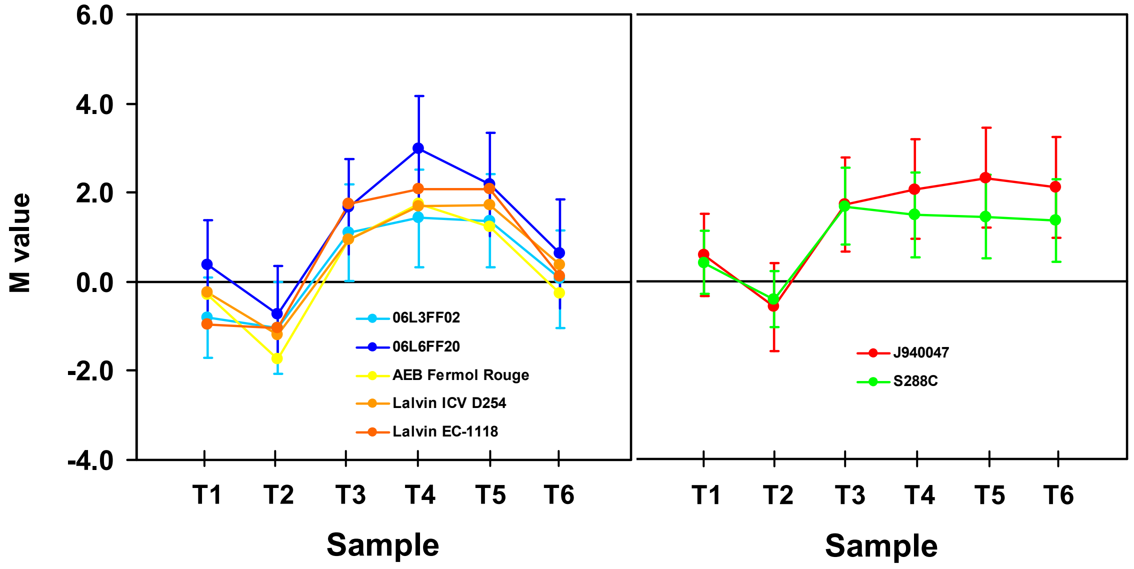

Supplemental Figure S2.
Mean relative transcript abundance of co-expressed genes in *S. cerevisiae* strains during fermentation in synthetic wine must. The relative transcript abundance was investigated for strains 06L3FF02, 06L6FF20, AEB Fermol Rouge, Lalvin ICV D254, Lalvin EC-1118, J940047 and S288C. Graphics represent the average relative transcript abundance (M value) calculated for clusters of genes with highly correlated expression profiles across samples. Standard error bars were only indicated for some strains, for clarity.

Cluster III: 841 genes

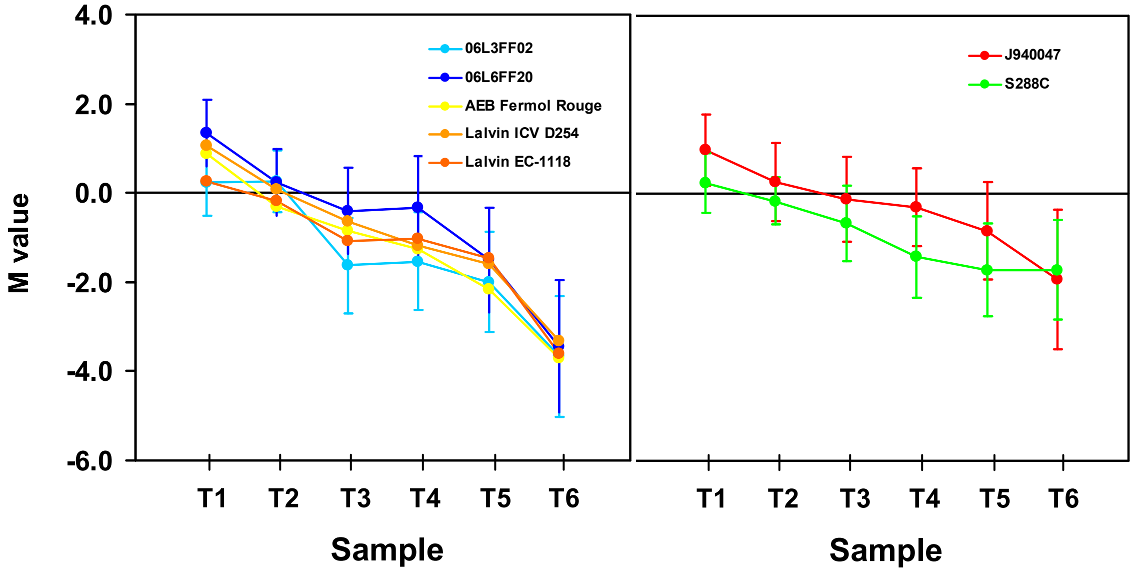

Cluster IV: 435 genes

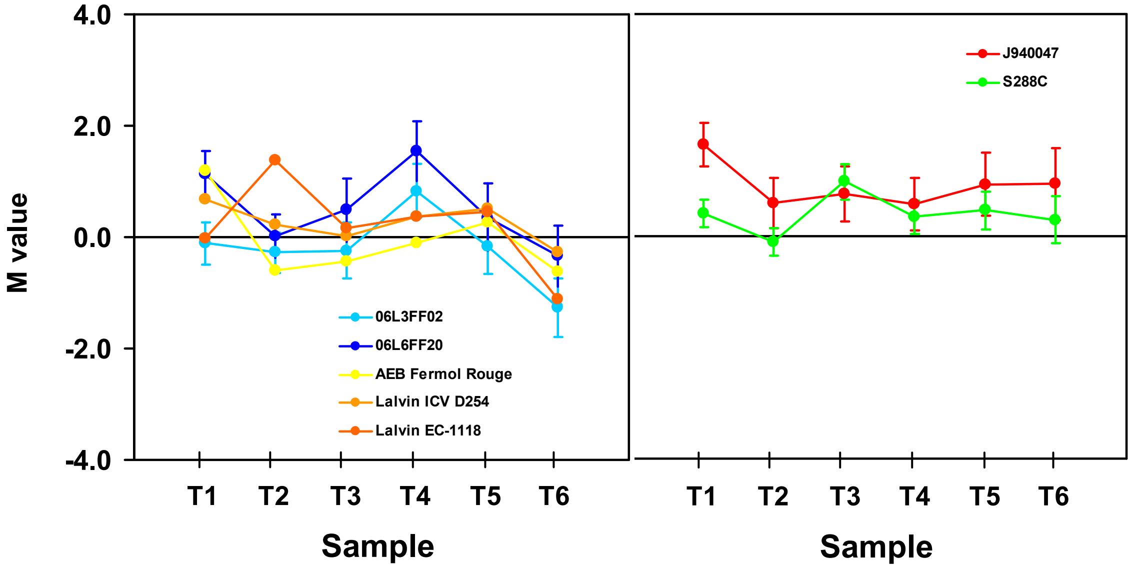

Supplemental Figure S2 (continued).
Mean relative transcript abundance of co-expressed genes in *S. cerevisiae* strains during fermentation in synthetic wine must.

Cluster V: 213 genes

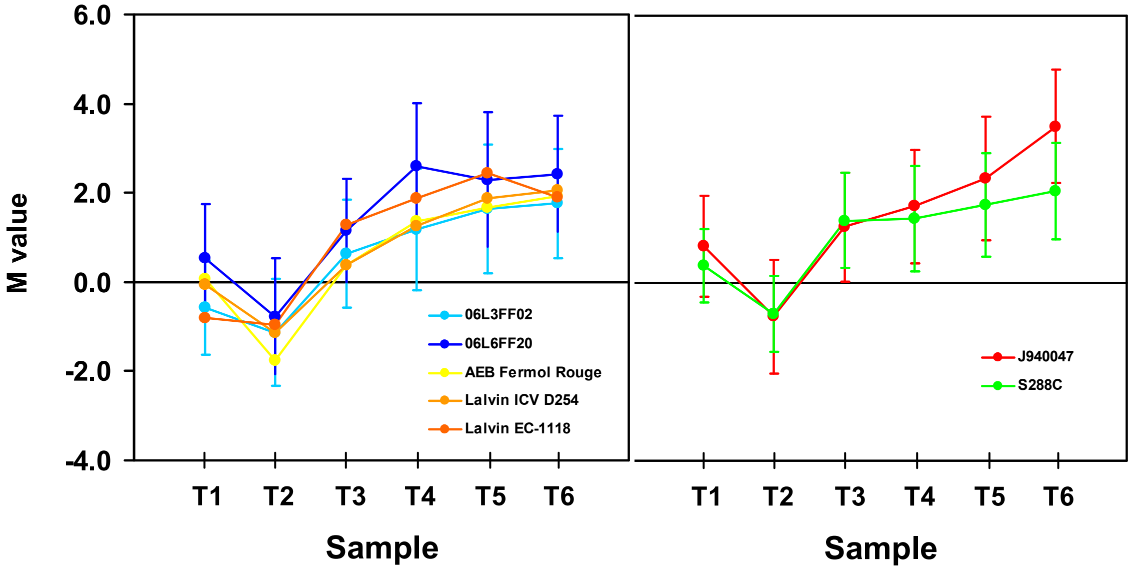

Cluster VI: 165 genes

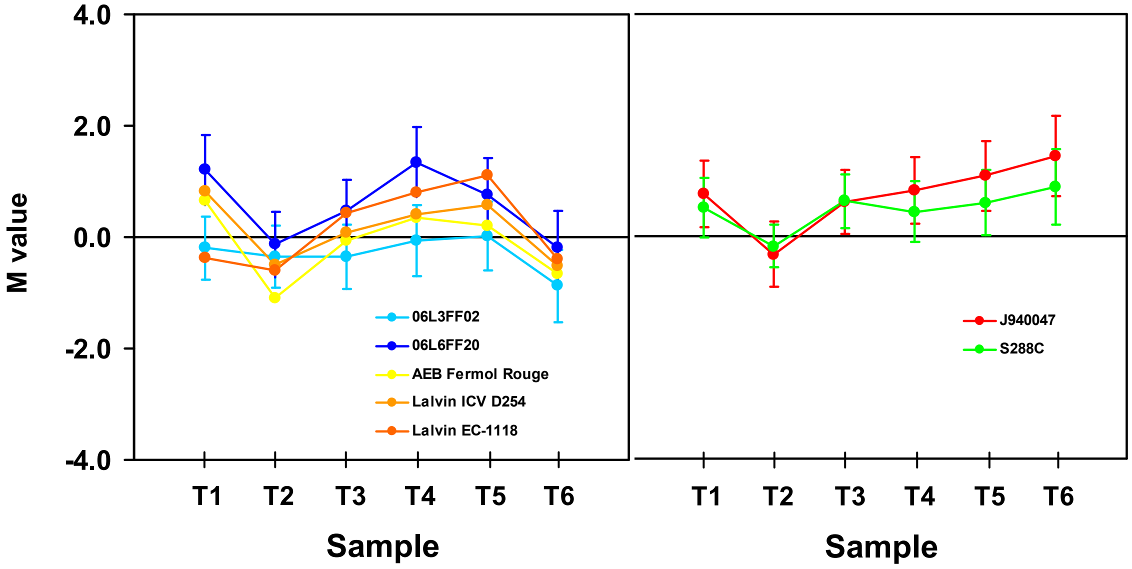

Supplemental Figure S2 (continued).
Mean relative transcript abundance of co-expressed genes in *S. cerevisiae* strains during fermentation in synthetic wine must.

Cluster VII: 128 genes

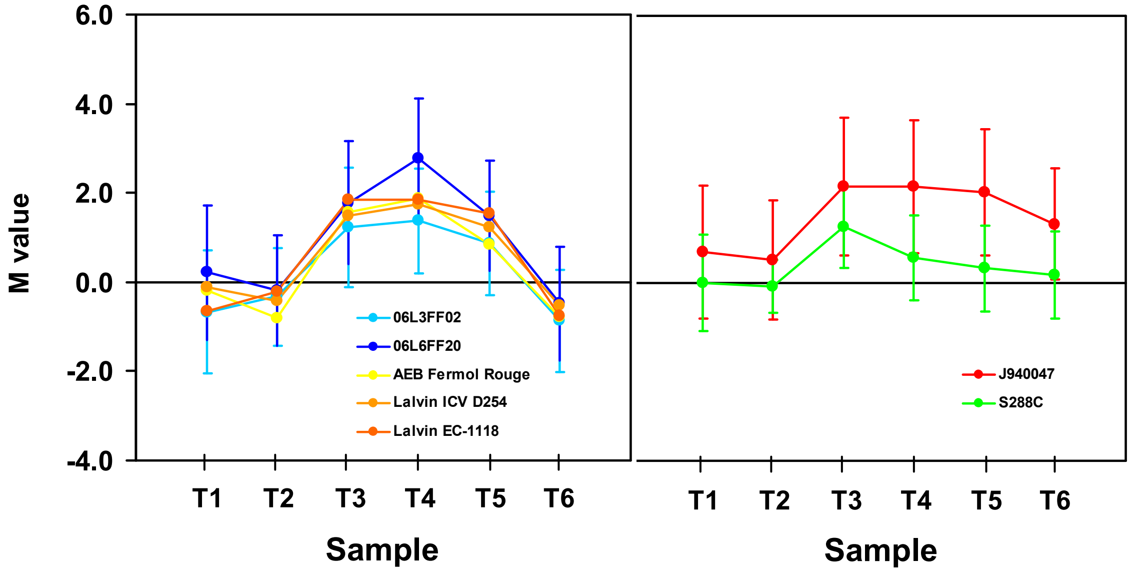

Cluster VIII: 109 genes

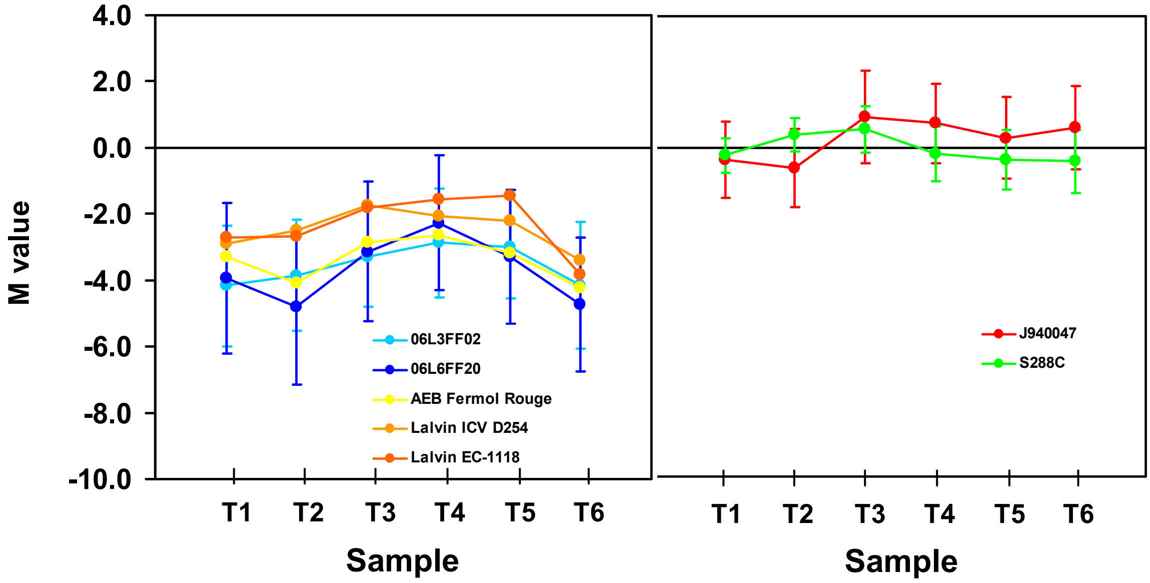

Supplemental Figure S2 (continued).
Mean relative transcript abundance of co-expressed genes in *S. cerevisiae* strains during fermentation in synthetic wine must.

Cluster IX: 103 genes

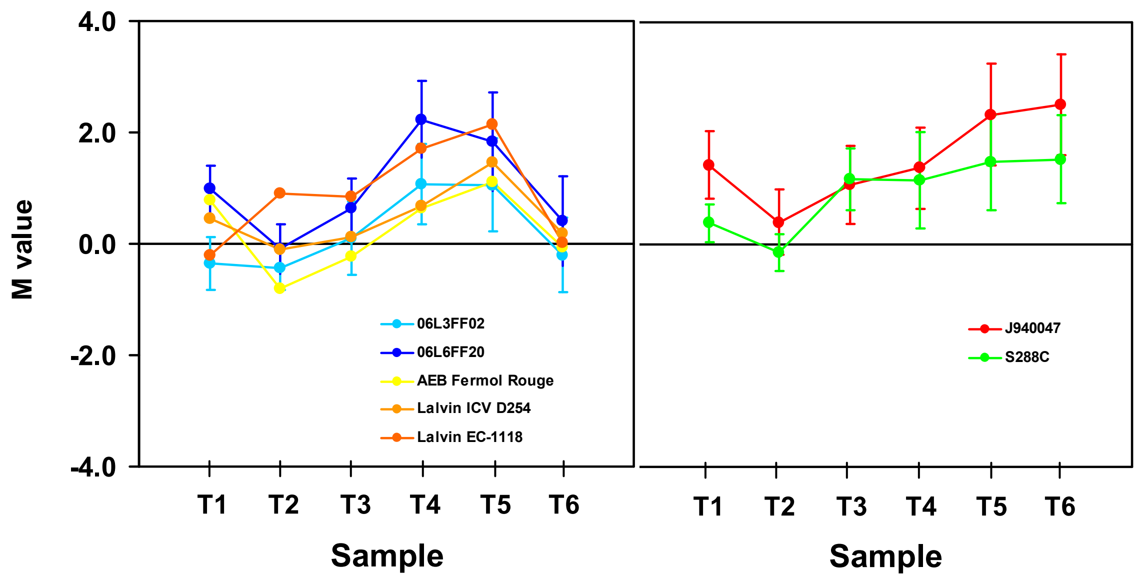

Cluster X: 79 genes

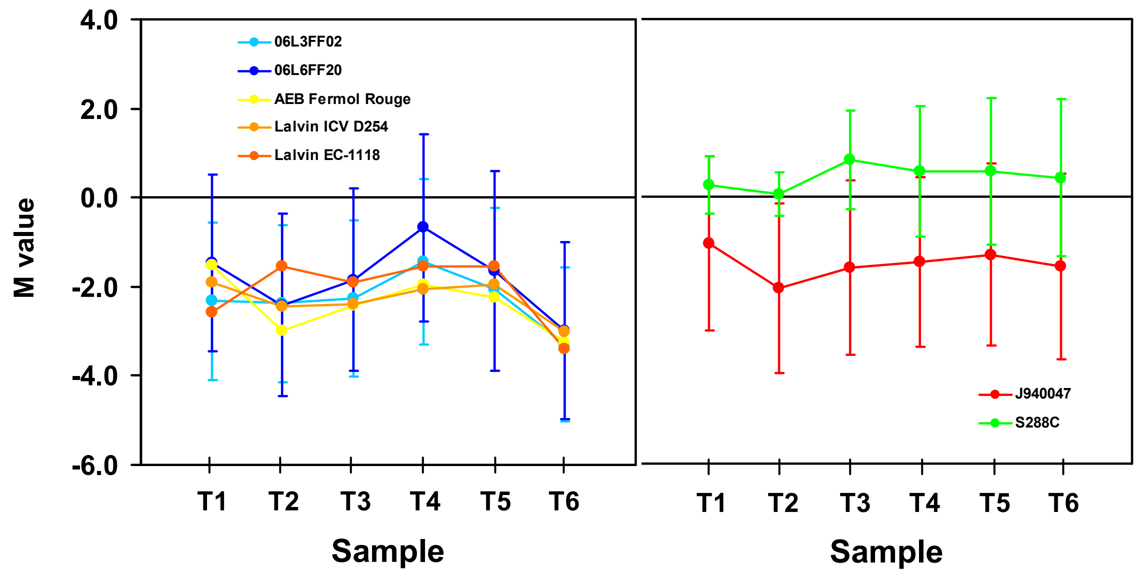

Supplemental Figure S2 (continued).
Mean relative transcript abundance of co-expressed genes in *S. cerevisiae* strains during fermentation in synthetic wine must.

Cluster XI: 73 genes

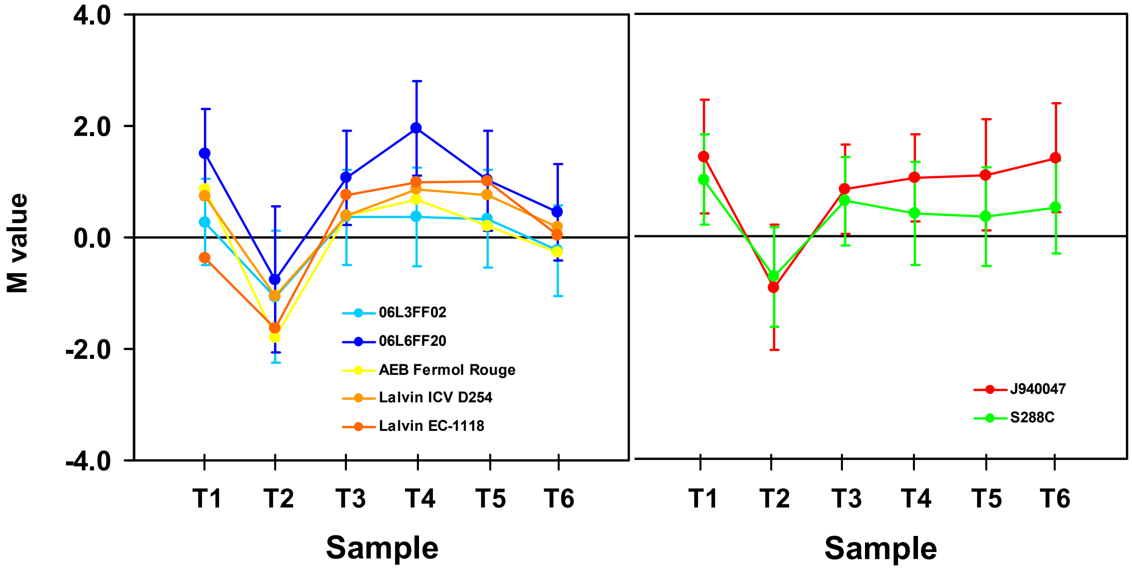

Cluster XII: 56 genes

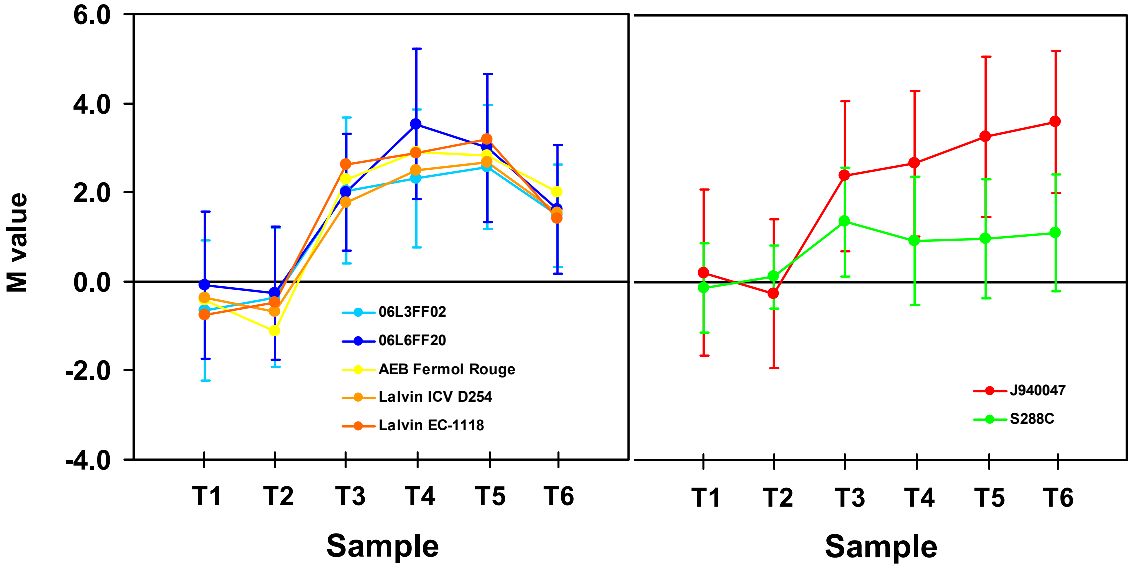

Supplemental Figure S2 (continued).
Mean relative transcript abundance of co-expressed genes in *S. cerevisiae* strains during fermentation in synthetic wine must.

Cluster XIII: 46 genes

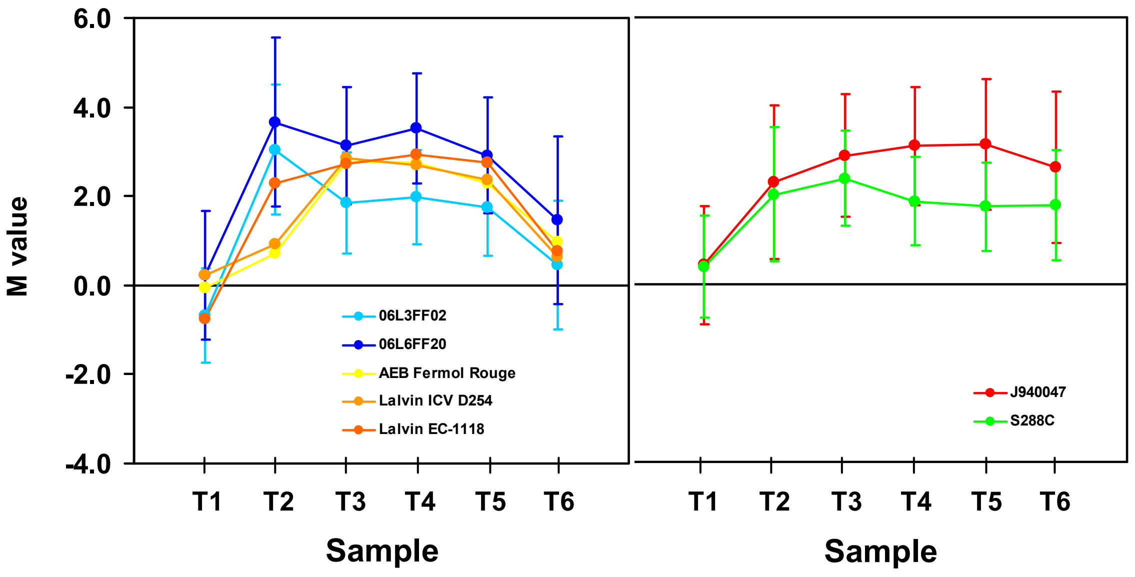

Low homogeneity cluster: 36 genes

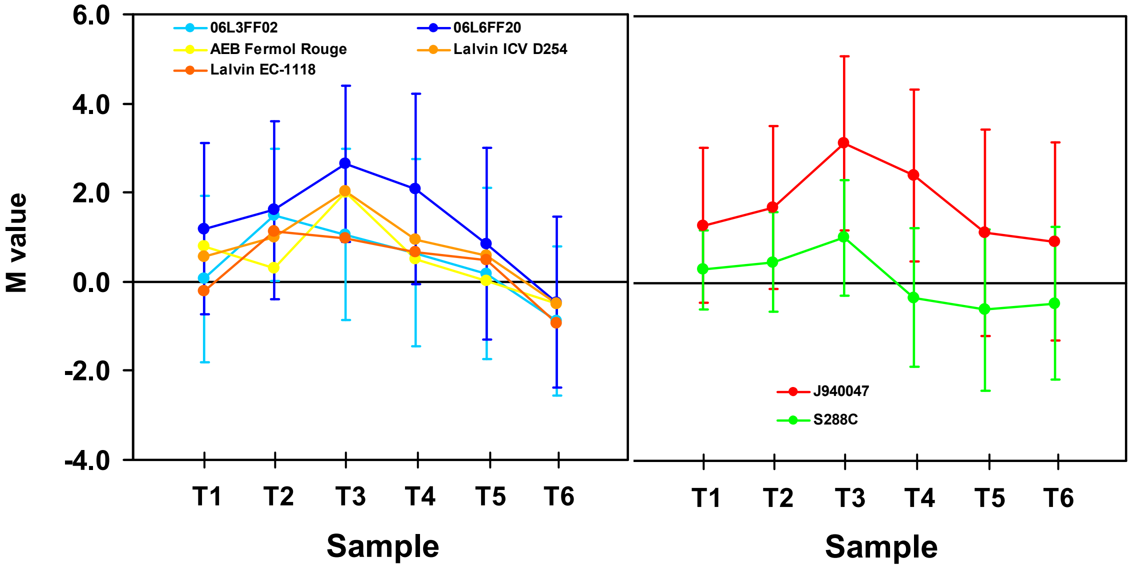

Supplemental Figure S2 (continued).
Mean relative transcript abundance of co-expressed genes in *S. cerevisiae* strains during fermentation in synthetic wine must.
