# Supplementary material for: Expression variability of co-regulated genes differentiates Saccharomyces cerevisiae strains
Source: BMC Genomics. 2011 Apr 20;12:201. doi: 10.1186/1471-2164-12-201 (PMC3094312; doi:10.1186/1471-2164-12-201)
Supplement: Additional file 3 — Relevant aCGH data. Summary of gene copy number differences observed between the Saccharomyces cerevisiae strains included in this study. Relative gene copy number values (fold change relatively to strain S288C) were obtained by comparative genome hybridization on array (Carreto et al. 2008. BMC Genomics 9: 524). Only genes relevant for discussion in the present manuscript were represented. [file 1471-2164-12-201-S3.PDF]

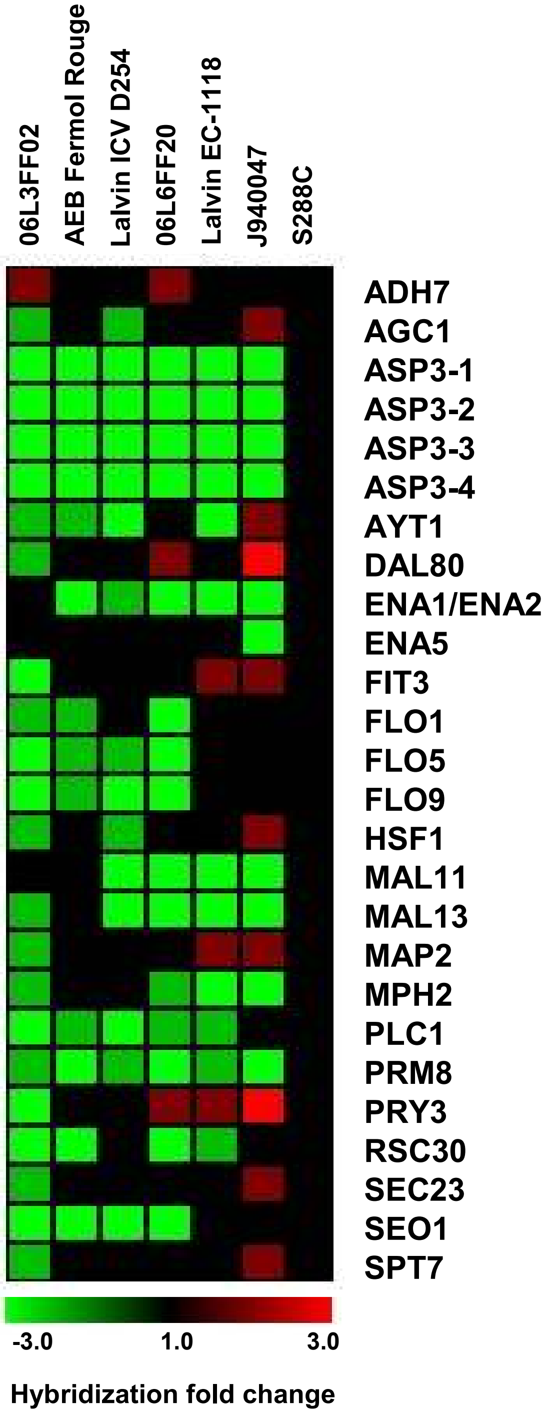

Supplemental Figure S3.
Gene copy number differences were detected among *S. cerevisiae* strains 06L3FF02, 06L6FF20, AEB Fermol Rouge, Lalvin ICV D254, Lalvin EC-1118 and J940047 relatively to the genome of strain S288C. Relative copy number values (fold change relatively to strain S288C) were obtained from a comparative genome hybridization on array (aCGH) study (Carreto *et al.* 2008. *BMC Genomics* 9: 524). Only the genes relevant for discussion in the present manuscript were represented.
