# Supplementary material for: Expression variability of co-regulated genes differentiates Saccharomyces cerevisiae strains
Source: BMC Genomics. 2011 Apr 20;12:201. doi: 10.1186/1471-2164-12-201 (PMC3094312; doi:10.1186/1471-2164-12-201)
Supplement: Additional file 4 — Variability in TATA box genes. Variability in gene expression was biased towards TATA box genes. The graphics show the frequency of TATA box genes as a function of gene expression variability. The average deviation from the mean of the relative gene expression value was taken as a measure of expression variability. Panel A was obtained considering all the investigated strains while Panel B represents the distribution obtained for the environmental and commercial strains. [file 1471-2164-12-201-S4.PDF]

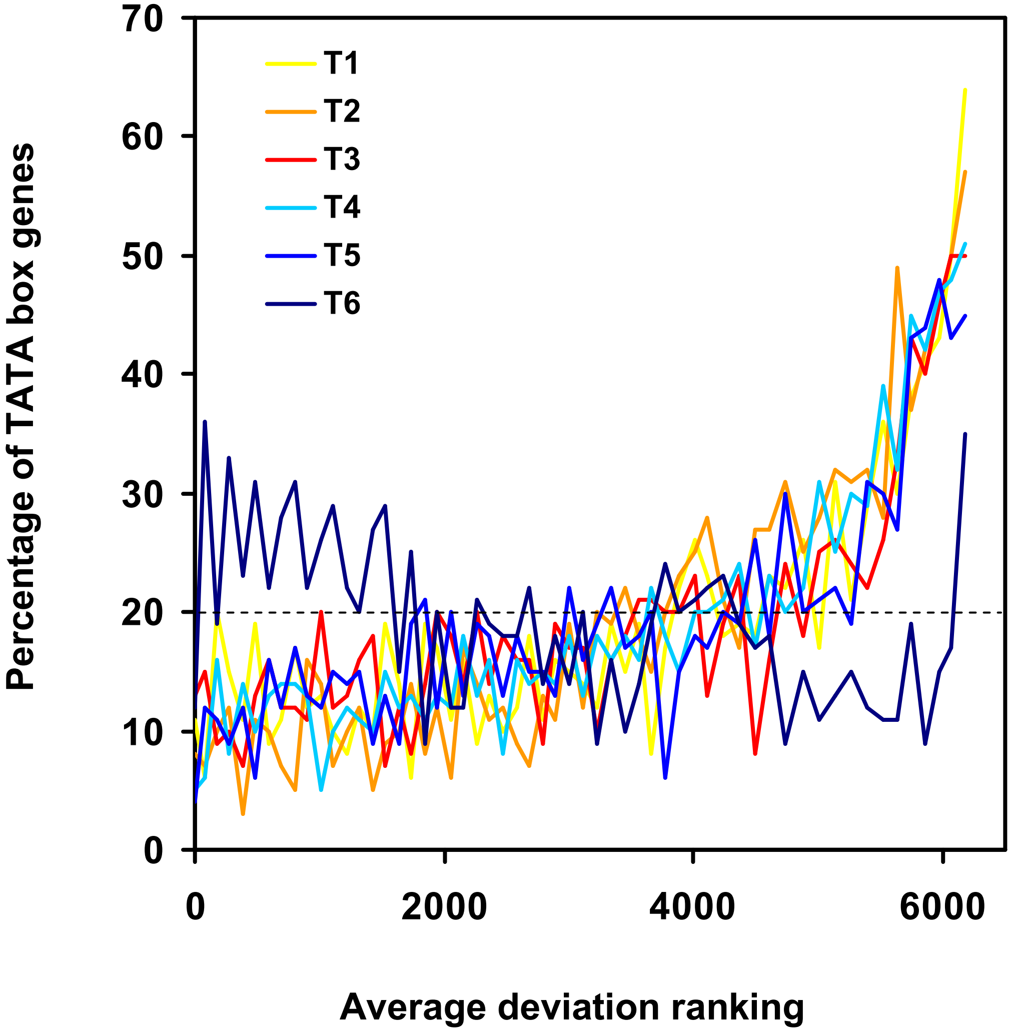

Supplemental Figure S4A.
Variability in gene expression was biased towards TATA box genes.
Transcriptome profiles were obtained for *Saccharomyces cerevisiae* strains 06L3FF02, 06L6FF20, AEB Fermol Rouge, Lalvin ICV D254, Lalvin EC-1118, J940047 and S288C during fermentation in synthetic wine must, at stages from T1 to T6 (see Manuscript for details). The average deviation from the mean relative transcript abundance of every examined ORF was taken as a measure of the respective variability in expression. The distribution of TATA box genes (per interval of 100 genes) was determined in a list of ORFs, ranked from the lowest to the highest average deviation value. TATA box genes were identified according to Basehoar *et al*., 2004. *Cell* 116: 699-709. For reference, the dashed line indicates the approximate percentage of TATA box genes in the genome of strain S288C.

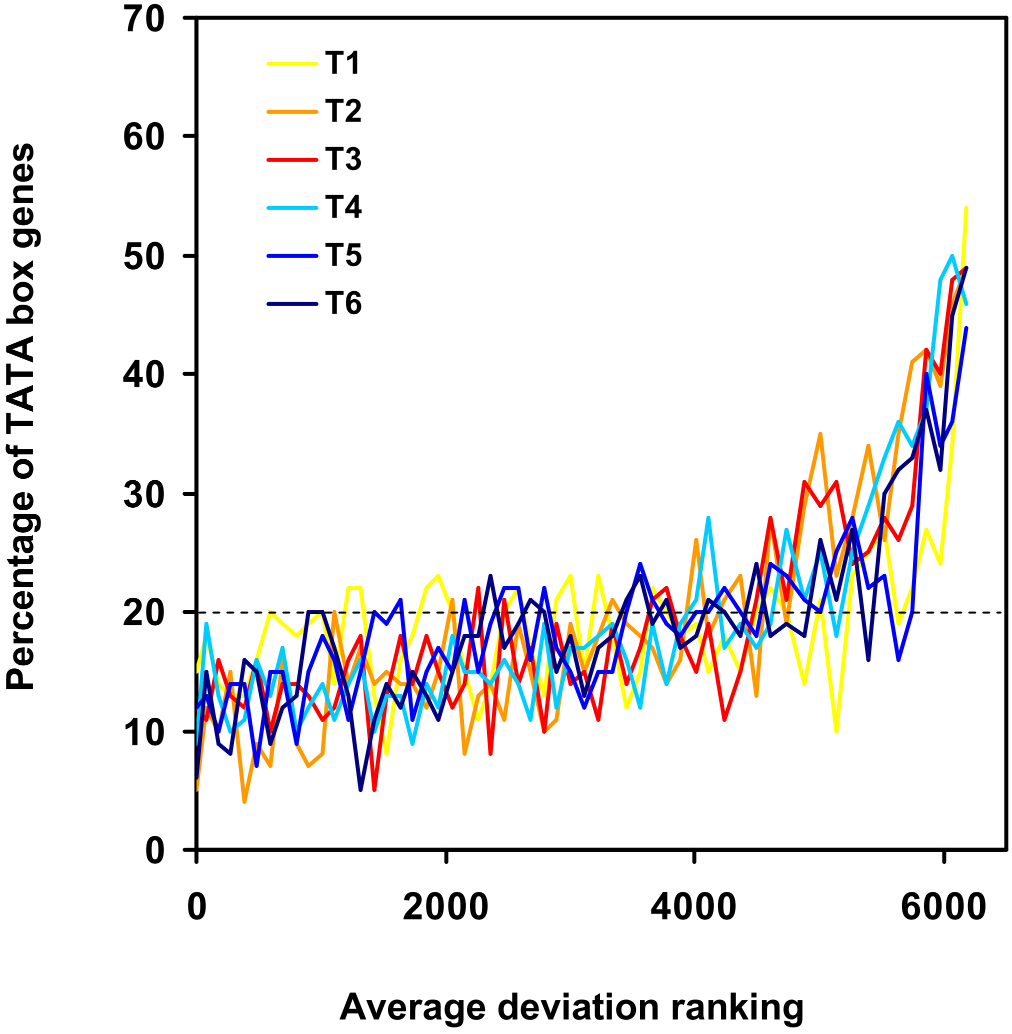

Supplemental Figure S4B.

Variability in gene expression was biased towards TATA box genes.

Analysis was performed only with wine strains 06L3FF02, 06L6FF20, AEB Fermol Rouge, Lalvin ICV D254, Lalvin EC-1118. Remaining legend as for Supplemental Figure S4A.
