# Supplementary material for: Expression variability of co-regulated genes differentiates Saccharomyces cerevisiae strains
Source: BMC Genomics. 2011 Apr 20;12:201. doi: 10.1186/1471-2164-12-201 (PMC3094312; doi:10.1186/1471-2164-12-201)
Supplement: Additional file 6 — Correlation between gene expression and aCGH. Correlation between relative transcript abundance and putative differences in gene load was found for some genes. The relative transcript abundance values determined for each strain relatively to those of strain S288C were plotted against the relative gene copy number differences for the same strains. The analysis was performed using the datasets from T2 (Panel A) and T4 (Panel B) stages of fermentation. [file 1471-2164-12-201-S6.PDF]

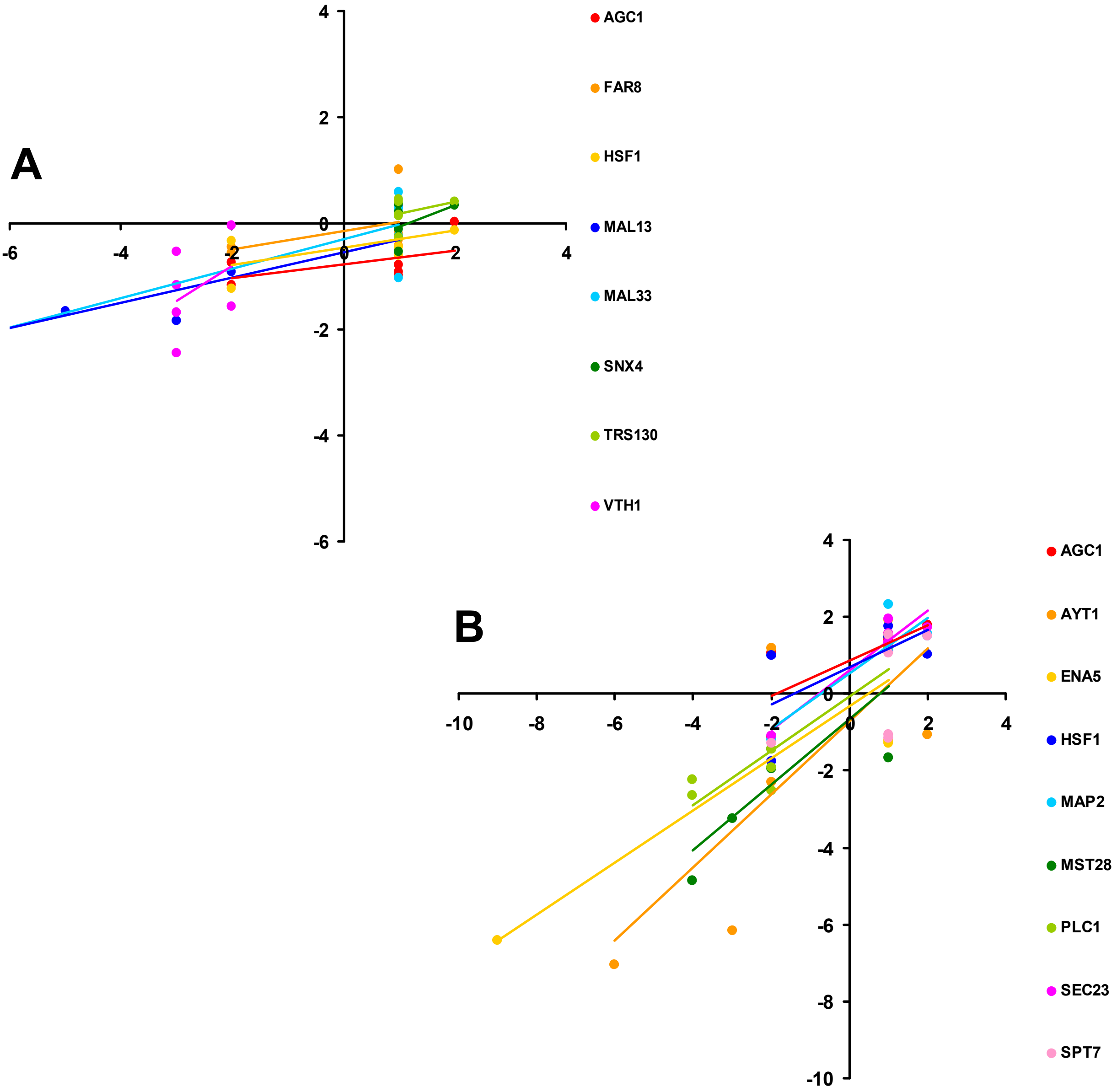

Supplemental Figure S5.

Correlation between relative gene expression and gene load among *S. cerevisiae* strains 06L3FF02, 06L6FF20, AEB Fermol Rouge, Lalvin ICV D254, Lalvin EC-1118, J940047 and S288C.

The relative transcript abundance values determined for each strain relatively to those of strain S288C were plotted against the relative gene copy number obtained from a previous genome comparison study (Carreto et al. 2008. BMC Genomics 9: 524). Only the genes within a two-fold deviation from absolute correlation in all the strains were represented. The analysis was performed using the datasets from the T2 (Panel A) and T4 (Panel B) stages of fermentation (see text for description). Colored dots identify the genes for which a correlation was found and the respective lines represent the average relationship between transcript abundance and gene load.
